# Downregulation of *MYPT1* increases tumor resistance in ovarian cancer by targeting the Hippo pathway and increasing the stemness

**DOI:** 10.1186/s12943-020-1130-z

**Published:** 2020-01-11

**Authors:** Sandra Muñoz-Galván, Blanca Felipe-Abrio, Eva M. Verdugo-Sivianes, Marco Perez, Manuel P. Jiménez-García, Elisa Suarez-Martinez, Purificacion Estevez-Garcia, Amancio Carnero

**Affiliations:** 1 Instituto de Biomedicina de Sevilla, IBIS, Hospital Universitario Virgen del Rocío, Universidad de Sevilla, Consejo Superior de Investigaciones Científicas, Avda. Manuel Siurot s/n 41013, Seville, Spain; 2 CIBERONC, Instituto de Salud Carlos III, Madrid, Spain

**Keywords:** Ovarian cancer, MYPT1 (*PPP1R12A)*, miR-30b, Therapy resistance, Hippo pathway, Stemness

## Abstract

**Background:**

Ovarian cancer is one of the most common and malignant cancers, partly due to its late diagnosis and high recurrence. Chemotherapy resistance has been linked to poor prognosis and is believed to be linked to the cancer stem cell (CSC) pool. Therefore, elucidating the molecular mechanisms mediating therapy resistance is essential to finding new targets for therapy-resistant tumors.

**Methods:**

shRNA depletion of *MYPT1* in ovarian cancer cell lines, miRNA overexpression, RT-qPCR analysis, patient tumor samples, cell line- and tumorsphere-derived xenografts, in vitro and in vivo treatments, analysis of data from ovarian tumors in public transcriptomic patient databases and in-house patient cohorts.

**Results:**

We show that *MYPT1* (*PPP1R12A*), encoding myosin phosphatase target subunit 1, is downregulated in ovarian tumors, leading to reduced survival and increased tumorigenesis, as well as resistance to platinum-based therapy. Similarly, overexpression of miR-30b targeting *MYPT1* results in enhanced CSC-like properties in ovarian tumor cells and is connected to the activation of the Hippo pathway. Inhibition of the Hippo pathway transcriptional co-activator YAP suppresses the resistance to platinum-based therapy induced by either low *MYPT1* expression or miR-30b overexpression, both in vitro and in vivo.

**Conclusions:**

Our work provides a functional link between the resistance to chemotherapy in ovarian tumors and the increase in the CSC pool that results from the activation of the Hippo pathway target genes upon *MYPT1* downregulation. Combination therapy with cisplatin and YAP inhibitors suppresses *MYPT1*-induced resistance, demonstrating the possibility of using this treatment in patients with low *MYPT1* expression, who are likely to be resistant to platinum-based therapy.

## Background

Ovarian cancer is the sixth most frequently occurring malignant tumor in women and the leading cause of death from gynecological malignancies worldwide [1]. The most frequent location of the tumor is the epithelium, and epithelial ovarian carcinoma is the most common form of the disease (approximately 90% of cases) [2]. Most advanced ovarian cancers are treated with a combination of debulking surgery and platinum-based chemotherapy, with cisplatin or its analogue carboplatin constituting first-line treatment. Although a significant proportion of patients initially respond to platinum-based treatment, most of these patients relapse in the next 18 months with a 5-year survival rate of approximately 30%. This relapse is mainly due to chemoresistance [3]. Therefore, it is essential to understand the resistance mechanisms and recover the response to treatment.

In recent years, cancer stem cells (CSCs) have emerged as major drivers of chemoresistance. CSCs are a subpopulation of cancer cells that possess the same self-renewal and differentiation capacities as stem cells, thereby maintaining tumor growth and the ability to regenerate a heterogeneous tumor mass [4, 5]. Thus, CSCs have been suggested to be responsible for metastasis and tumor growth and development [6–8]. Furthermore, it has been reported that traditional chemotherapy fails to target CSCs, which could account for relapse [7]. Therefore, it is feasible that the CSCs that reside in ovarian epithelial tumors are not targeted by chemotherapy and are responsible for chemotherapy failure.

The Hippo pathway is a regulator of tissue growth and cell fate that is evolutionarily conserved from flies to humans. This pathway consists of a large network of proteins that control tissue growth during development and differentiation but also in pathological situations, such as cancer [9]. The core pathway consists of a kinase cassette that is composed of the mammalian sterile 20-like kinases (MST1/2) and the large tumor suppressor kinases (LATS1/2) [10]. NF2 (called Merlin in *Drosophila*) is responsible for the pathway activation through MST1/2 phosphorylation. NF2/Merlin is dephosphorylated and inactivated by PP1a, the heterodimer formed by the catalytic subunit PPP1Ca and its targeting and regulatory protein MYPT1. MYPT1 belongs to the family of myosin phosphatase targeting proteins (MYPT) and plays a role in the regulation of smooth muscle contraction [11, 12], but other functions of MYPT1 have been discovered recently, such as migration and cell adhesion [13], cell cycle [14, 15] and development [16]. The main Hippo core kinase cascade includes the mammalian transcriptional co-activator Yes-associated protein (YAP) and its paralog transcriptional co-activator with the PDZ-binding motif (TAZ). The phosphorylation of YAP and TAZ by the Hippo pathway leads to their sequestration in the cytoplasm and ubiquitination-dependent proteasomal degradation [17].

In many tumors, upon Hippo signaling inhibition, YAP and TAZ translocate into the nucleus to promote cell proliferation in cooperation with transcription factors, such as TEAD, SMADs, RUNXs, p63/p73, PAX3, PPARc, TTF1 and TBX-5. These transcription factors regulate target genes that are involved not only in cell proliferation but also in tissue growth, the control of organ size and shape and metastasis [18–22]. In mice, mutations in the Hippo pathway leading to YAP or TAZ hyperactivation cause cell proliferation and promote pluripotency and dedifferentiation [23, 24]. Accordingly, it has been reported that YAP acts as an oncogene and has been associated with poor prognosis in ovarian cancer [8, 25, 26]. When MYPT1 binds to the phosphatase PP1, the specificity of MYPT1 for different substrates increases [27, 28]. MYPT1-PP1 was shown to dephosphorylate Merlin/NF2 at serine 518, thereby leading to the activation of the kinase cascade that leads to YAP/TAZ inhibition [29] and preventing tumor progression [30]. Therefore, MYPT1 is a key regulator of the Hippo pathway.

Our work provides a functional link between the resistance to chemotherapy in ovarian tumors and the increase in the CSC pool that results from the inhibition of the Hippo pathway upon *MYPT1* downregulation. Combination therapy with cisplatin and YAP inhibitors suppresses *MYPT1*-induced resistance, demonstrating the possibility of using this treatment in patients with low *MYPT1* expression, who are likely to be resistant to platinum-based therapy.

## Methods

### Cell culture

Cells were cultured according to the manufacturer’s recommended procedure in McCoy (ES-2 line) or RPMI (SKOV3 and OVCAR8 lines) and incubated at 37 °C in 5% CO_2_ in a humidified atmosphere. Parental cells ES-2, SKOV3 and OVCAR8 were obtained from ATCC.

### Gene transfer

It was performed as previously described [31]. The shRNA *PPP1R12A* (*MYPT1*) and miRNA-30b were provided by Origene.

### Proliferation assay

It was performed as previously described [32].

### Cytotoxic MTT assay

A total of 5 × 10^3^ ES-2, SKOV3 or OVCAR8 cells were seeded and then treated with platinum drugs and/or YAP inhibitor (verteporfin) 24 h later. After 96 h, cell viability was measured with MTT.

### Luciferase assay

For assaying the transcriptional repressive capacity of miR-30b, we cloned a fragment of the 3′-UTR of *MYPT1* gene into the pmirGLO vector (Promega) using primers 5′-ATCGACGGAGCTCTGCAGCTGCTGAGAAGATTT-3′ and 5′-CGTCGATTCTAGACGAAACTGTGGCACATCAAA-3′, containing SacI and XbaI sites, respectively. Luciferase assay was performed with the Dual-Luciferase Reporter Assay System (Promega) following the manufacturer’s instructions.

### Maintenance of mouse colonies

All experiments involving animals received expressed approval from the IBIS/HUVR Ethical Committee for the Care and Health of Animals. They were maintained in the IBIS animal facility according to the facility guidelines, which are based on the Real Decreto 53/2013 and were sacrificed by CO_2_ inhalation, either within a planned procedure or as a human endpoint when the animals showed significant signs of illness.

### In vivo xenograft studies

Tumor growth was assayed by the subcutaneous injection of 4 × 10^6^ SKOV3 or OVCAR8 cells that were transfected with a shRNA against *MYPT1* in cohorts of five nude mice each that were analyzed weekly. Tumors were measured using calipers. All mice were sacrificed once the growth experiment was completed.

### In vivo xenograft treatment

Tumors were harvested when they reached 1500 mm^3^, cut into 2 × 2 × 2 mm pieces and re-implanted. Mice were randomly allocated to the drug-treated and control-treated (solvent only) groups, and once the tumor reached 20 mm^3^, the mice received the appropriate treatment for 4 weeks (2 doses/week). Mice were monitored daily for signs of distress and weighed twice a week. The tumor size was measured, and the size was estimated according to the following equation: tumor volume = [length x width^2^]/2. The experiments were terminated when the tumor reached 350 mm^3^ or when the clinical endpoint was reached. The drugs cisplatin and carboplatin were obtained from pharmacy HUVR and were freshly prepared and administered by intraperitoneal injection. We used higher doses in mice, assuming a 70 kg average weight for humans (in humans is 125 mg/dose) [33]. We administered two doses per week: 3.5 mg/kg per dose for cisplatin and 15 mg/kg per dose for carboplatin (equivalent to 7 mg/kg and 30 mg/kg, averaging 25 g body weights for each mouse). We did not observe signs of toxicity.

### Colony formation assay and clonal heterogeneity analysis

A total of 10^3^ cells were seeded onto 10 cm plates, and every condition was evaluated in triplicate. The medium was replaced every 3 days for 12 days, and the colonies were fixed, stained and counted. Values are expressed as the number of observed colonies among the 10^3^ seeded cells. To analyze the clonal heterogeneity, 10^2^ random colonies were classified in triplicate as having the following phenotypes: holoclone, meroclone and paraclone [34].

### Sphere-forming assay

A total of 10^3^ cells were resuspended in 1 ml of complete MammoCultTM Basal Medium (Stemcell Tech) and seeded in ultralow attachment plates. Cultures were imaged, the tumorspheres were counted, and their diameters were quantified using the CellSenseDimension software on days 2, 3 and 4.

### In vivo xenografts from tumorspheres

It was assayed by the subcutaneous injection of 10^3^ cells grown as tumorspheres into the hind legs of 4-week-old female athymic nude mice. Animals were treated as describe previously, examined twice a week and incubated for 4 weeks more, then killed and tumors extracted. Tumors were measured using calipers.

### Immunohistochemistry

Tumor samples were obtained at HUVR by ovarian cancer patients by surgical resection and stored in TMA blocks. Samples from our xenografts were also stored in TMA blocks. Immunohistochemistry assays were performed as previously described [35], with minor modifications. Blinded evaluation of high or low signal intensity was performed by semiquantitative microscopic analysis.

### Western blot analyses

Western blotting was performed according to standard procedures. The primary antibodies and dilutions were used as indicated in Additional file 1: Table S1.

### RT–qPCR

Total RNA was isolated using an RNeasy kit (Qiagen), and cDNA was generated from 1 μg of RNA with MultiScribe Reverse Transcriptase (Applied Biosystems). The qPCR reaction was performed using a TaqMan Assay (Applied Biosystems) with probes as indicated in Additional file 1: Table S1. Relative mRNA expression was calculated as 2^-∆Ct^.

### Taqman Array

To analyze the expression levels of genes of the Hippo signaling pathway, we used the human TaqMan Array Human Hippo Signaling Pathway 96-well fast plates (Applied Biosystems), with cDNA obtained as detailed above and following manufacturer’s recommendations. Data were analyzed in a ViiA 7 qPCR system (Applied Biosystems). Heatmaps, representing either z-scores or expression fold-changes relative to the empty vector-expressing cells, were done with the Multiexperiment Viewer software (https://sourceforge.net/projects/mev-tm4/). Hierarchical clustering of samples were performed by the complete linkage method according to a Pearson’s correlation.

### Fluorescence-activated cell sorting

For FACS staining, live cells were incubated with antibodies for 30 min at dilutions specified in the manufacturer’s protocols. See Additional file 1: Table S1.

### Quantification and statistical analysis

All statistical analyses were performed using GraphPad Prism 4. The distribution of quantitative variables among different study groups was assessed using parametric (Student’s *t*-test) or nonparametric (Kruskal–Wallis or Mann–Whitney) tests, as appropriate. Experiments were performed a minimum of three times and were performed in independent triplicates each time. Survival data from patient databases were analyzed by the Log-rank Mantel-Cox statistical test.

### Analyses of cancer patient databases

We performed meta-analyses of the public patient datasets from the R2 Genomics analysis and visualization platform (http://hgserver1.amc.nl) to analyze the *MYPT1* expression levels in tumor and non-tumor ovarian samples from the databases. Statistical significance of the tumor versus normal samples was assessed (*P* < 0.05). Correlation between miRNA expression levels and *MYPT1* expression was analyzed using the TCGA ovarian database (www.cbioportal.org [36]). Patient survival was analyzed using the PrognoScan public patient datasets (http://dna00.bio.kyutech.ac.jp/PrognoScan/index.html). Kaplan-Meier plots showing patient survival were generated using databases with available survival data with the scan method, which searches for the optimum survival cut-off based on statistical analyses (log-rank test), thereby identifying the most significant expression cut-off.

### Patient cohort

The entire procedure was approved by the local ethical committee of the HUVR (CEEA O309-N-15). A cohort of paraffin-embedded tissue samples from 22 patients with ovarian cancer was obtained from the biobank of Hospital Universitario Virgen del Rocío-Instituto de Biomedicina de Sevilla (Sevilla, Spain) for RNA expression studies and for the evaluation of the correlation of clinicopathological features. Samples were obtained from biopsies of patients who had been subjected to platinum treatment and who were evaluated for their response according to RECIST criteria; normal tissue, platinum-resistant tumor samples and platinum-sensitive tumor samples were obtained. Tumor samples were sent to the pathology laboratory for diagnosis and were prepared for storage with formalin fixation and paraffin embedding. Samples were stained with hematoxylin/eosin, and RNA was extracted from the tumor tissue.

## Results

### *MYPT1* is downregulated in ovarian tumors and is associated with reduced overall survival

**Fig. 1:**
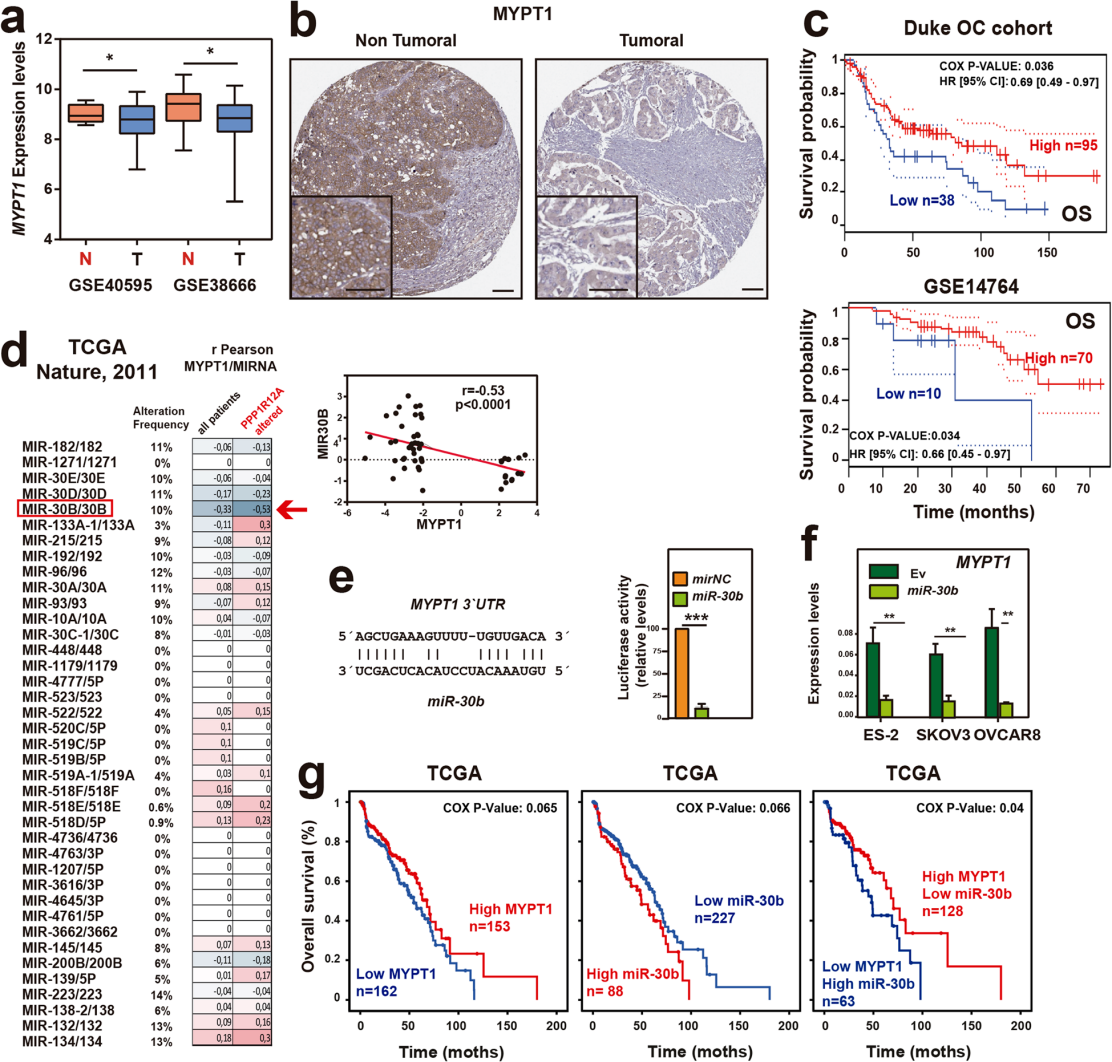
*MYPT1* is downregulated by miR-30b in ovarian tumors and reduces overall survival in ovarian cancer patients. **a** *MYPT1* expression in the GSE40595 and GSE38666 ovarian cancer patient databases. Box plots showing the expression levels of *MYPT1* in ovarian tumor tissue (blue) or non-tumor tissue (red) patients. Data were analyzed by comparing the tumor versus the normal samples using Student’s *t-*test. *, *P* < 0.05. **b** Representative images of MYPT1 immunostaining in ovarian cancer and non-tumoral ovary samples. **c** Kaplan-Meier plots showing overall survival of patients with high (red) or low (blue) *MYPT1* expression levels in two databases with survival data (Duke OC cohort and GSE14764). Data were analyzed with the log-rank test, and the associated *P*-values are shown in the graphs. **d** Correlation of the expression levels of miRNAs and *MYPT1* in the TCGA ovarian cancer database. Data were analyzed using Pearson’s R correlation. *, *P* < 0.05; **, *P* < 0.01; ***, *P* < 0.001. **e** *Left*, putative miR-30b binding site in the 3′ -UTR of the *MYPT1* gene. *Right*, luciferase activity assay of the 3′-UTR of *MYPT1* in HEK293 cells expressing or not (mirNC) miR-30b. **f** *MYPT1* expression levels measured by RT-qPCR in ES-2, SKOV3 or OVCAR8 ovarian cancer cell lines expressing miR-30b or EV. **g** Kaplan-Meier plots showing overall survival of patients with high (red) or low (blue) *MYPT1* expression levels (left), high (red) or low (blue) *miR-30b* expression levels (middle) or their combination (right) in the TCGA ovarian cancer database. Data were analyzed with the log-rank test, and the associated *P*-values are shown in the graphs

To study the possible role of MYPT1 in ovarian cancer, we first analyzed the *MYPT1* expression levels in two public ovarian cancer databases that contain both normal and tumor samples, GSE40595 and GSE38666 (Additional file 1: Table S2). We found that the *MYPT1* mRNA levels were significantly lower in tumor samples than in normal ovarian tissue (Fig. 1a). This data was corroborated at the protein level by analyzing the expression levels of *MYPT1* in tumor and normal samples by immunohistochemistry (Fig. 1b). To test whether decreased *MYPT1* expression had any relevance to the survival of patients, we plotted the survival probabilities of those patients with low or high levels of *MYPT1* expression using data from the DUKE OC and GSE14764 databases. We found that patients with lower *MYPT1* expression showed a significant decrease in survival in the analyzed databases compared to patients with higher *MYPT1* expression (Fig. 1c). These results suggest that *MYPT1* could act as a tumor suppressor in ovarian cancer.

### Expression of the microRNA miR-30b is inversely correlated with *MYPT1* expression

Since microRNAs (miRNAs) are commonly deregulated in cancer and may play a role in regulating the expression of oncogenes and tumor suppressor genes, we investigated whether the expression of *MYPT1* could be regulated by specific miRNAs. To this end, we first examined the TCGA database [36] for miRNAs whose expression was correlated with that of *MYPT1* in ovarian cancer patients. We analyzed this correlation in either total patients or only those showing deregulated *MYPT1* expression and selected miRNAs showing higher correlation in the second case (Fig. 1d). We found that miR-30b expression, which was deregulated in 10% of ovary tumors, fitted this condition and showed the highest negative correlation with *MYPT1* expression (r = − 0.53, *p* < 0.0001; Fig. 1d). Additionally, we found a target sequence of miR-30b in the 3′-UTR of the *MYPT1* gene (Fig. 1e), suggesting that this miRNA could directly target *MYPT1*. To confirm this, we first analyzed the capacity of miR-30b to block *MYPT1* expression by cloning a fragment of *MYPT1* 3′-UTR containing the putative miR-30b target sequence into a luciferase reporter vector, finding that miR-30b expression lead to a large decrease in luciferase activity (Fig. 1e). Then, we overexpressed miR-30b in three ovarian cancer cell lines (ES-2, SKOV3 and OVCAR8) and measured *MYPT1* expression levels by RT-qPCR. We observed a large reduction of the mRNA transcript of *MYPT1* in cells ectopically overexpressing miR-30b (Fig. 1f). Finally, analysis of the TCGA database showed us that 80.7% of ovarian cancer patients had copy number alterations of the miR-30b gene, being 24.4% amplifications that were related with a significantly higher expression of the gene (Additional file 3: Figure S1).

Then, we examined whether miR-30b expression was related to patient survival. To test this possibility, provided that the databases used above do not contain miRNA expression data, we analyzed the TCGA cohort. First, we corroborated that low *MYPT1* expression levels were correlated with worse survival in this patient cohort (Fig. 1g). Then, we analyzed the relevance of miR-30b expression for patient survival and found that those expressing high levels of miR-30b showed lower survival probabilities (Fig. 1g). Finally, the combination of both miR-30b and *MYPT1* expression clearly showed that patients with statistically significant lower survival probabilities were those with combined lower *MYPT1* expression and higher miR-30b expression. This suggests that miR-30b could be downregulating *MYPT1* expression and that low *MYPT1* expression leads to decreased survival of ovary cancer patients either by itself or by upregulation of miR-30b expression.

### Decreased *MYPT1* expression leads to Hippo pathway deactivation in ovary cancer cell lines

**Fig. 2:**
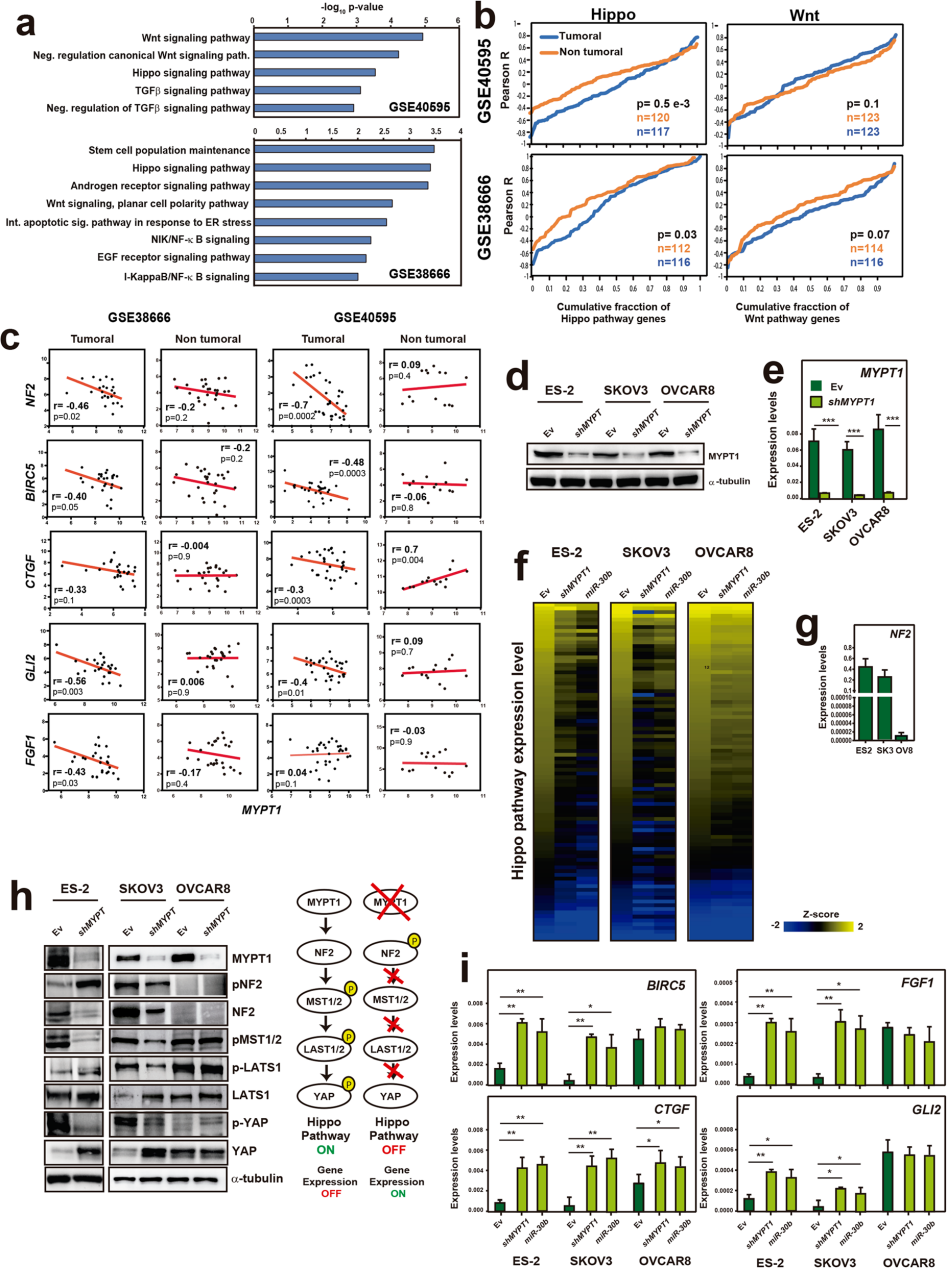
Downregulation of MYPT1 leads to Hippo pathway deactivation in ovarian cancer. **a** Gene Ontology term enrichment analyses of the genes whose expression levels correlated with the levels of *MYPT1* in the GSE40595 and GSE38666 databases. Only biological process terms involving signaling pathways were selected. **b** Cumulative distribution of the Pearson’s correlation in the Hippo (left) or Wnt (right) pathway genes from GSE40595 (top) or GSE38666 (bottom). **c** Correlation of the expression levels of the *NF2* and Hippo targets *BIRC5, CTGF, GLI2* and *FGF1* with the expression levels of *MYPT1* in the GSE40595 and GSE38666 databases, in tumoral and non-tumoral tissue. Data were analyzed using Pearson’s R correlation. *, *P* < 0.05; **, *P* < 0.01; ***, *P* < 0.001. **d** Western blot showing the protein levels of MYPT1 in ES-2, SKOV3 and OVCAR8 cells expressing EV or *shMYPT1*. **e** Analysis of the *MYPT1* expression level by RT-qPCR in ES-2, SKOV3 or OVCAR8 cells expressing EV or *shMYPT1*. **f** Heatmaps showing the z-scores of Hippo pathway gene expression obtained from TaqMan Array Human Hippo Signaling Pathway 96-well fast plates containing probes against Hippo pathway genes. Genes are sorted according to decreasing z-scores in the EV-expressing cells independently for each cell line. **g** Analysis of the *NF2* expression level by RT-qPCR in ES2, SKOV3 or OVCAR8 cells. **h** *Left*, western blot showing the activation status of the Hippo signaling pathway in SKOV3 or OVCAR8 ovarian cancer cells expressing *shMYPT1* or EV. Protein levels of pNF2 (S518), NF2, pMST1/2 (T180/183), pLATS1 (T1079), LATS, pYAP (S127), YAP, MYPT1 and α-tubulin are shown. *Right*, scheme showing the main components of the Hippo pathway and their activity with and without MYPT1. **i** Analysis of the expression of several Hippo pathway target genes, including *BIRC5*, *CTGF*, *FGF1* and *GLI2*, by RT-qPCR in ES-2, SKOV3 or OVCAR8 ovarian cancer cells expressing *shMYPT1,* miR-30b or EV

To gain insight into the molecular mechanism connecting the *MYPT1* expression levels with tumorigenesis, we searched for genes whose expression correlated with that of *MYPT1* in tumor samples from the databases GSE40595 and GSE38666. We found that 7222 and 6197 genes correlated with *MYPT1*, respectively (*P* < 0.05). Gene Ontology (GO) term enrichment analyses of these genes showed a variety of enriched biological processes (Additional file 2: Dataset), among which we identified some terms related to signaling pathways that are involved in tumorigenesis (Fig. 2a and Additional file 2: Dataset). Only two of these signaling pathways were found in both databases: the Wnt and Hippo pathways (Fig. 2a). To determine whether these pathways could collectively correlate with *MYPT1* in tumor samples, we evaluated the correlations between *MYPT1* expression in each database and every gene annotated in these pathways. We found that negative correlations with the Hippo pathway genes were significantly more negative in tumor samples than in normal tissue, which was not observed for the Wnt pathway genes (Fig. 2b-c). Therefore, these data suggest a role for Hippo in *MYPT1*-induced tumorigenesis.

It has been shown that MYPT1 is a regulatory subunit of the PP1A enzyme, which targets NF2, whose dephosphorylation at serine 518 is the initial step in the Hippo pathway, resulting in growth arrest and tumor suppression [29, 30]. To study the role of MYPT1 and miR-30b during ovarian tumorigenesis, we generated three ovarian cell lines, ES-2, SKOV3 and OVCAR8, that were *MYPT1-*depleted (two independent *shMYPT1* constructs were analyzed, but only one is shown in the main figures; see the Additional Files for the results obtained with the other *shMYPT1* construct), expressed miR-30b or an empty vector (EV). Both *shMYPT1* and miR-30b expression led to downregulated expression of *MYPT1* (Fig. 2d-e, Fig. 1f and Additional file 3: Figure S2a). To assess the activity of the Hippo pathway in these ovarian tumor cells and the effect of *MYPT1* downregulation, we first measured the expression levels of Hippo pathway genes by RT-qPCR using custom TaqMan Array plates containing probes against Hippo pathway genes (Fig. 2f and Additional file 2: Dataset). We found a general decrease in Hippo pathway gene expression in cells expressing either *shMYPT1* or miR-30b, which was clear in ES-2 and SKOV3 cells but very slight in OVCAR8 cells (Fig. 2f). Notably, the fold-change in expression of these genes was highly correlated between *shMYPT1-* and miR-30b-expressing cells for all three cell lines (Additional file 3: Figure S2b), suggesting that the effect of miR-30b expression is mediated by *MYPT1* downregulation. The lack of effect in the OVCAR8 cell line was intriguing, and we observed a general decrease in Hippo pathway gene expression in EV-expressing cells compared with ES-2 and SKOV3 cells (Additional file 3: Figure S2c). Indeed, NF2 expression was considerably lower in OVCAR8 cells than in ES-2 and SKOV3 cells, as determined by RT-qPCR (Fig. 2g), confirming the constitutive downregulation of Hippo pathway gene expression in this cell line.

Next, to determine whether the results at the transcript level were related to protein activity, we analyzed the protein levels and phosphorylation status of the main Hippo pathway proteins (NF2, MST1/2, LATS1/2 and YAP) in our cell lines expressing EV or *shMYPT1*. We found that both ES-2 and SKOV3 cells expressing *shMYPT1* showed a less active Hippo pathway with an increased ratio of phospho-NF2/total NF2 compared to those of the EV-expressing cells (Fig. 2h and Additional file 3: Figure S2d). Accordingly, the *MYPT1*-depleted cells showed reduced phospho-MST1/2 and phospho-LATS1 levels, as well as reduced phospho-YAP (Ser127) and increased total YAP levels (Fig. 2h and Additional file 3: Figure S2a). We also analyzed the levels of YAP and TAZ in the cytoplasmic and nuclear fractions and found that YAP and TAZ localization to the nucleus were increased upon *MYPT1* downregulation (Additional file 3: Figure S2e). These results indicate that the Hippo pathway activity is decreased upon *MYPT1* downregulation, leading to increased translocation of its transcriptional effector YAP to the cell nucleus. In contrast, OVCAR8 cells expressed minimal levels of NF2/Merlin, even in cells expressing the EV, leading to a constitutively decreased activity of the Hippo pathway and subsequent YAP dephosphorylation (Fig. 2h and Additional file 3: Figure S2a). Accordingly, the low level of NF2 in OVCAR8 cells was associated with the specific methylation of the *NF2* gene promoter in these cells (Additional file 3: Figure S2f).

Then, to confirm the activation status of the Hippo pathway in these conditions, we measured the expression levels of several Hippo target genes, including *BIRC5, CTGF, FGF1* and *GLI2*. We found that the expression of these target genes was increased in the *MYPT1*-depleted ES-2 and SKOV3 cells compared to the EV (Fig. 2i and Additional file 3: Figure S1 g), confirming that the pathway was inactivated and therefore allowed YAP-mediated target gene expression. Accordingly, target gene expression in OVCAR8 cells was higher even in the EV-expressing cells and was not further increased in most cases upon *MYPT1* downregulation, confirming the constitutive inactivation of the Hippo pathway in these cells. Finally, expression of miR-30b led to similar effects in target gene expression as *shMYPT1* expression in all three cell lines (Fig. 2i), reinforcing the notion that the miR-30b effect is mediated by *MYPT1* downregulation.

### Downregulation of *MYPT1* increases tumor growth in ovarian cancer cells

**Fig. 3:**
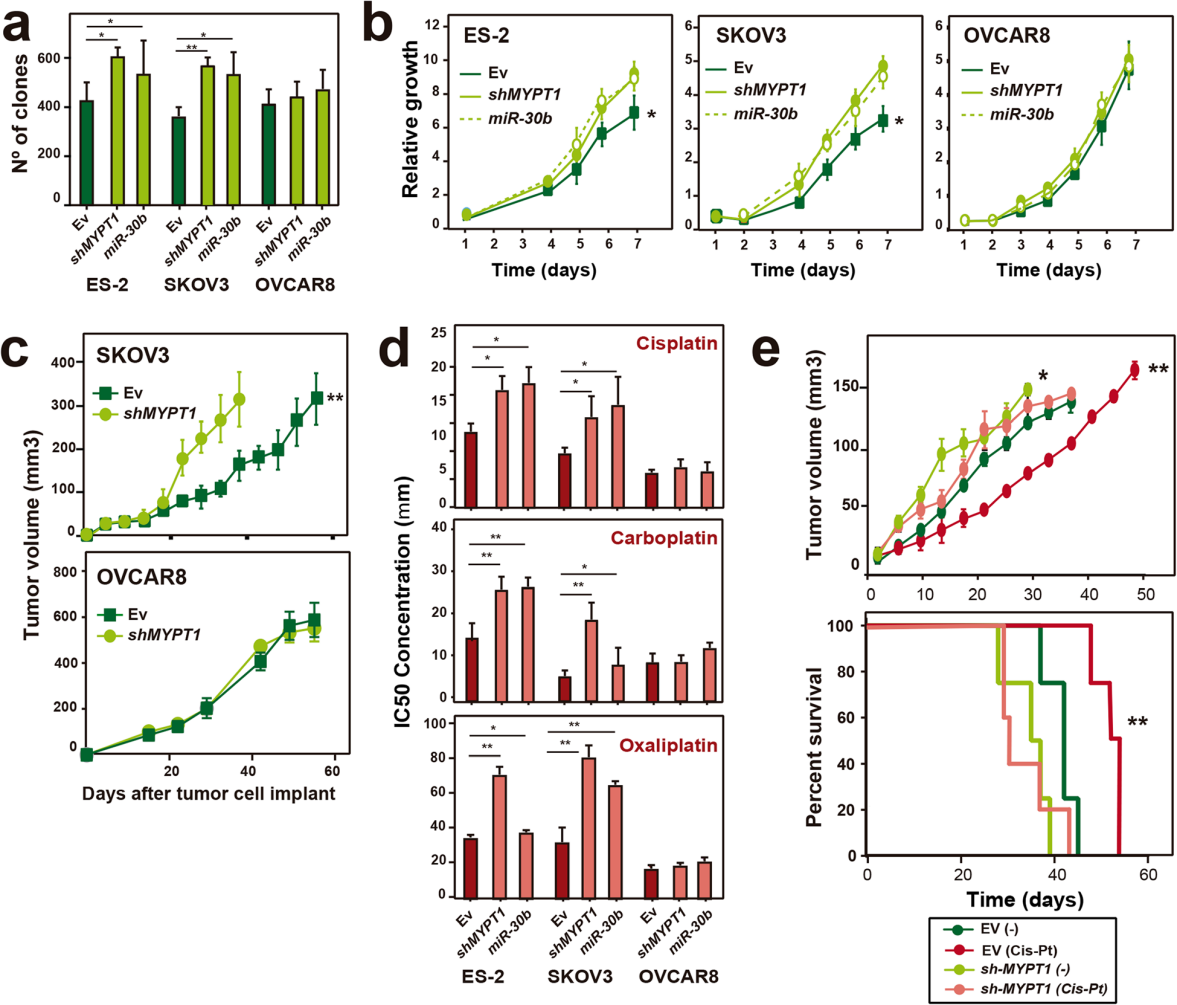
Downregulation of *MYPT1* increases tumorigenesis and resistance to platinum-based therapy in ovarian cancer cells in vivo and in vitro. **a** Quantification of the number of clones in the ES-2, SKOV3 or OVCAR8 ovarian cell lines expressing an EV (dark green), *shMYPT1* or miR-30b (light green). **b** Growth curve of the ES-2, SKOV3 and OVCAR8 ovarian cell lines expressing an EV (dark green), *shMYPT1* or miR-30b (light green) represented as doubling times. **c** Tumor growth in xenografts from SKOV3 and OVCAR8 cell lines expressing an EV (dark green) or *shMYPT1* (light green), which were injected into female athymic nude mice (4 × 10^6^ cells/ mouse). Cohorts of 5 mice each were used. **d** Determination of the IC50 (concentration of drug necessary to induce 50% cell death) for platinum drugs in cells overexpressing *shMYPT1*, miR-30b (light red) or EV (dark red). **e** Determination of the tumor volume and survival after cisplatin treatment in xenografts of SKOV3 cells expressing *shMYPT1* or EV. Cohorts of 5 mice each were either treated with cisplatin or saline once the tumor reached 0.5 cm in diameter, and the survival rates were determined. All experiments were repeated at least three times. Data were analyzed using Student’s *t-*test. *, *P* < 0.05; **, *P* < 0.01; ***, *P* < 0.001

The association of low levels of *MYPT1* expression with poor patient survival prompted us to analyze whether *MYPT1* downregulation affected tumorigenesis. To this end, we first examined the ability of cells to form colonies at low density. We observed a significant increase in the number of colonies that were formed by ES-2 and SKOV3 cells, but not by OVCAR8 cells, upon *MYPT1* downregulation compared to those of the EV cells (Fig. 3a; Additional file 3: Figure S3a). Expression of miR-30b led to a similar effect. Accordingly, we found that *shMYPT1*- and miR-30b-expressing ES-2 and SKOV3 cells grew faster than control cells (EV), while both vector-, shRNA- and miR-30b-expressing OVCAR8 cells grew quickly (Fig. 3b; Additional file 3: Figure S3b).

Next, to determine whether *MYPT1* expression had any effect on tumor progression in vivo, we generated xenografts with SKOV3 or OVCAR8 cells that were overexpressing either EV or *shMYPT1* and injected into animal cohorts. We found that the animals that were injected with the cells with low *MYPT1* levels showed enhanced tumor growth compared to that of the controls only in the SKOV3-derived xenografts (Fig. 3c). However, the OVCAR8-derived xenografts grew at the same rate when they were generated with either EV or *shMYPT1*-expressing cells. Interestingly, immunostaining of the xenografts showed that NF2 levels are lower in *MYPT1*-depleted SKOV3-derived tumors, but constitutively low in OVCAR8-derived ones, and that YAP translocates to the nucleus in SKOV3-derived tumors upon *MYPT1* depletion, while it is constitutively nuclear in OVCAR8-derived ones (Additional file 3: Figure S4). These results corroborate that *MYPT1* downregulation increases tumor growth in vivo only in cells in which the Hippo pathway is not constitutively inactive (Fig. 2), suggesting that *MYPT1* depletion contributes to tumorigenesis through inactivation of the Hippo pathway.

### Downregulation of *MYPT1* increases resistance to platinum therapy in ovarian tumors

Ovarian cancer is the type of gynecological tumor that causes the most deaths, most of them as a result of relapse or resistance to treatment, usually cisplatin or its analogue carboplatin. We therefore examined whether the reduction of *MYPT1* expression in ovarian cancer increases resistance to platinum-based therapies. We first subjected cells to different doses of platinum drugs to calculate the IC50 in vitro. We found that ES-2 and SKOV3 ovarian tumor cells expressing either *shMYPT1* or mir-30b were more resistant with a 2- to 3-fold higher IC50 for platinum drugs (cisplatin, carboplatin and oxaliplatin) than that of control cells (Fig. 3d and Additional file 3: Figure S3c). In contrast, OVCAR8 cells depleted of *MYPT1* (with *shMYPT1* or mir-30b) had IC50 values for platinum drugs that were similar to those of the control cells (Fig. 3d and Additional file 3: Figure S3c).

To confirm these data in vivo, we generated xenografts with SKOV3 cells expressing EV or *shMYPT1*. Each cohort of mice was treated with either cisplatin or saline solution once their tumors reached a diameter of 0.5 cm. As expected, cisplatin treatment caused a 40% reduction of tumor volume compared to that of the control in xenografts generated with SKOV3 parental cells (Fig. 3e), increasing the survival time by more than 20% (53 vs. 42 days, respectively; Fig. 3e). In contrast, cisplatin treatment did not have any effect on xenografts that were generated from *shMYPT1*-expressing SKOV3 cells (Fig. 3e) in comparison with xenografts that were generated with EV-expressing SKOV3 ovarian cells. Moreover, these mice showed a 15% reduction in survival compared to that of untreated mice (Fig. 3e). Similar but more modest results were observed with carboplatin treatment (Additional file 3: Figure S3d). Taken together, these data indicate that the depletion of *MYPT1* induces resistance to platinum drugs both in vitro and in vivo.

### Reduced expression of *MYPT1* leads to increased stemness in ovarian cancer cells

**Fig. 4:**
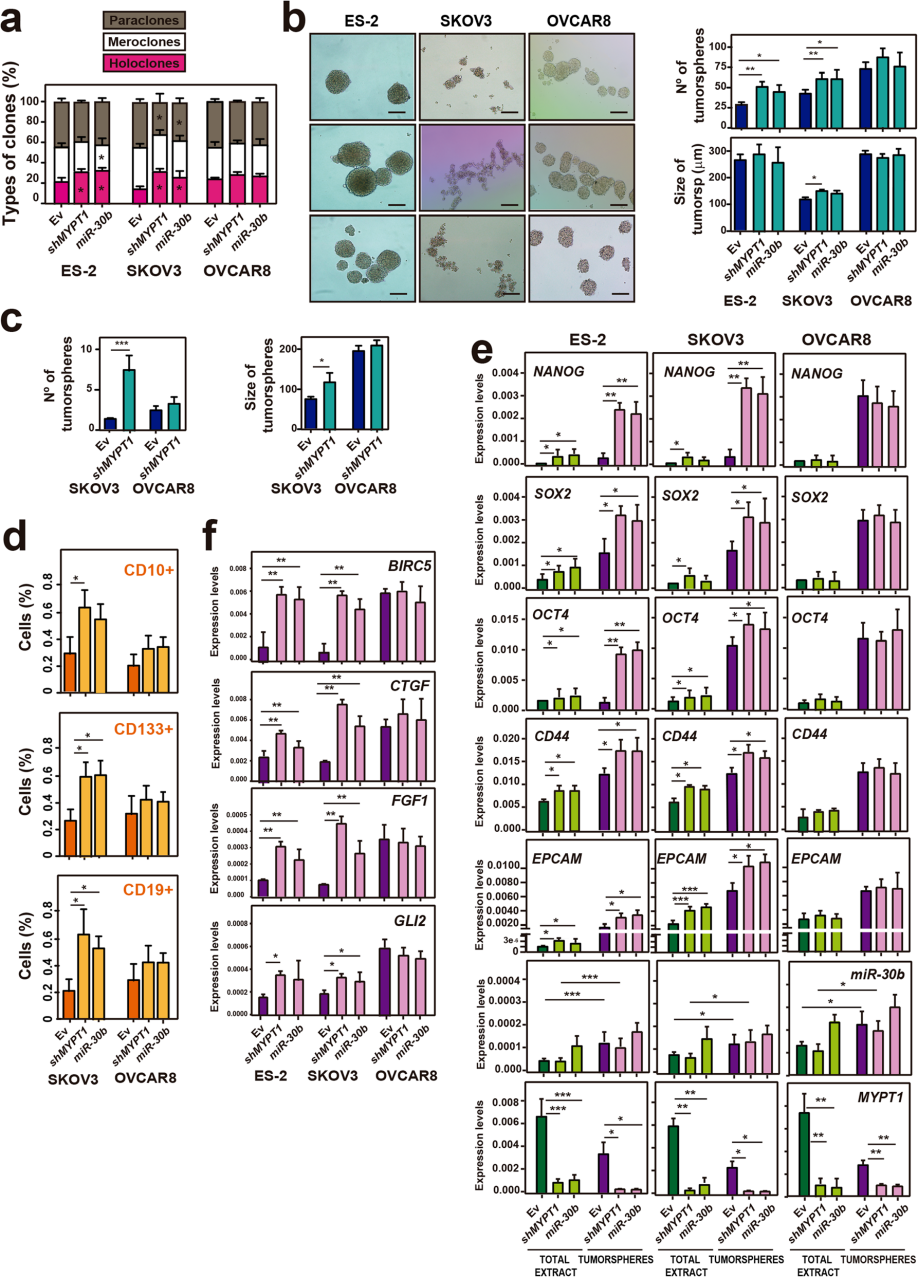
Downregulation of *MYPT1* increases stemness in ovarian cancer cells. **a** Percentage of paraclones, meroclones and holoclones generated by ES-2, SKOV3 or OVCAR8 ovarian cells expressing *shMYPT1*, miR-30b or EV. **b** *Left,* representative images of tumorspheres formed by ES-2, SKOV3 and OVCAR8 cells expressing *shMYPT1,* miR-30b or EV. Scale bars: 100 μm. *Right,* quantification of the number and size of tumorspheres. **c** Quantification of the number and size of tumorspheres formed by SKOV3 and OVCAR8 cells expressing *shMYPT1* or EV from single cells. **d** Quantification of the percentage of cells that were CD10+, CD133+ or CD19+ (CSC surface markers) by FACS. **e** Analysis of the expression by RT-qPCR of the stemness-associated genes *OCT4, NANOG* and *SOX2*, the CSC-related genes *CD44* and *EPCAM,* as well as *MYPT1* and miR-30b, in total cell extracts and tumorspheres from ES-2, SKOV3 or OVCAR8 ovarian cancer cells expressing *shMYPT1*, miR-30b or EV. **f** Analysis of the expression of several Hippo pathway target genes, including *BIRC5, CTGF, FGF1* and *GLI2*, by RT-qPCR in tumorspheres from ES-2, SKOV3 or OVCAR8 ovarian cancer cells expressing *shMYPT1*, miR-30b or EV. The averages and SDs of three independent experiments are shown. Data were analyzed using Student’s *t-*test. *, *P* < 0.05; **, *P* < 0.01; ***, *P* < 0.001

Provided that resistance to therapy in tumors has been attributed to CSCs, we explored whether decreased *MYPT1* expression could increase the stem-cell features of ovarian cancer cells. To this end, we first grew individual ES-2, SKOV3 or OVCAR8 cells expressing *shMYPT1*, miR-30b or EV and analyzed the formation of holoclones, meroclones and paraclones (Fig. 4a and Additional file 3: Figure S5a), which are different types of colonies that are believed to be formed by stem cells, transit-amplifying cells and differentiated cells, respectively [37]. We found that the depletion of *MYPT1*, either mediated by *shMYPT1* or miR-30b expression, led to a significant increase in the percentage of holoclones and a decrease in the percentage of paraclones in ES-2 and SKOV3 cells but not in OVCAR8 cells (Fig. 4a and Additional file 3: Figure S5a). To further assess this phenomenon, we analyzed the formation of tumorspheres, which are enriched in CSCs. We found that a reduction of *MYPT1* expression caused a significant increase in the number of tumorspheres formed specifically by ES-2 and SKOV3 cells, but not by OVCAR8 cells, while an increase in the size of tumorspheres was also detected for SKOV3 cells (Fig. 4b and Additional file 3: Figure S5b). These results were corroborated by analyzing the formation of tumorspheres from single cells (Fig. 4c and Additional file 3: Figure S5c). These data indicate that *MYPT1* downregulation increases the stemness of ovarian cancer cells and suggest an increased population of CSCs in these conditions.

Next, we performed FACS analyses to measure the expression of a variety of CSC surface markers, including *CD10, CD19, CD24, CD34, CD44, CD117, CD133* and *CD184,* in ovarian tumor cells (Additional file 1: Table S3). We observed that *MYPT1* depletion and miR-30b expression led to a significant increase in CD10+, CD133+ and CD19+ SKOV3 cells but not in OVCAR8 cells (Fig. 4d and Additional file 3: Figure S6). CD24, CD44 and CD184 were not increased upon *MYPT1* downregulation (Additional file 1: Table S3). Additionally, we analyzed the expression levels of stemness-associated genes, including *OCT4, NANOG* and *SOX2*, in total cell extracts and in tumorspheres from ES-2, SKOV3 and OVCAR8 cells. We found that both the depletion of *MYPT1* or the expression of miR-30b led to a significant increase in the expression levels of stem genes in total extracts of the ES-2 and SKOV3 cells, whereas we did not observe an increase in OVCAR8 cells (Fig. 4e and Additional file 3: Figure S5d). Moreover, we measured the expression levels of the CSC markers *CD44* and *EPCAM* [38–40], showing also an upregulation only in *MYPT1-*depleted ES-2 and SKOV3 cells (Fig. 4e). We observed similar results in tumorspheres from these cell lines but with higher stem gene expression (Fig. 4e and Additional file 3: Figure S5d). These results reinforce the idea that *MYPT1* downregulation increases the stemness of ovarian cancer cells specifically in those cells where the Hippo pathway is active.

According to the presented data, we reasoned that if MYPT1 acts as a tumor suppressor that regulates the stem-like properties of ovarian cancer, then we should observe low expression levels of *MYPT1* in tumorspheres compared to those in total cell extracts. To evaluate this hypothesis, we analyzed the expression levels of *MYPT1* in total extracts and tumorspheres from ES-2, SKOV3 and OVCAR8 cells. Our results showed that tumorspheres had lower expression levels of *MYPT1* than those in total cell extracts from the three ovarian tumor cell lines (Fig. 4e; Additional file 3: Figure S3e). Consistently with our model of *MYPT1* regulation by miR-30b, tumorspheres from ovarian tumor cell lines also showed increased miR-30b expression, reinforcing the miR-30b-MYPT1 axis as an important regulator of stemness (Fig. 4e). Taken together, our results demonstrate that *MYPT1* downregulation leads to an increase in stem-like properties and confirm that MYPT1 is a tumor suppressor in ovarian cancer.

### *MYPT1* downregulation in ovarian cancer cells induces stemness properties by targeting the Hippo pathway

To study whether the activity of the Hippo pathway could be related to the stem-like properties that are induced by *MYPT1* downregulation, we used RT-qPCR to analyze the expression levels of different Hippo pathway target genes in tumorspheres derived from ES-2, SKOV3 and OVCAR8 cells expressing EV, *shMYPT1* or miR-30b. Tumorspheres derived from ES-2 or SKOV3 cells showed increased expression of Hippo target genes, including *BIRC5*, *CTGF*, *FGF1* and *GLI2*, upon *shMYPT1* or miR-30b expression (Fig. 4f). Moreover, we noted that the expression levels of the Hippo targets in OVCAR8 cells were higher than those in ES-2 and SKOV3 cells, including in control cells, and that these levels remained high in tumorspheres that were generated from these cells (Fig. 4f and Fig. 2i) because of the constitutive inactivation of the Hippo pathway in OVCAR8 cells. Therefore, these data could explain the differences that were observed in tumorigenesis and in the induction of stem-like properties between SKOV3 and OVCAR8 cells that were expressing or not expressing *shMYPT1*. Altogether, these results strongly suggest that the lower levels of *MYPT1* induced a deactivation of the Hippo pathway and that this phenomenon is consistent with an increase in the CSC pool.

### YAP inhibition suppresses resistance to platinum treatment in *MYPT1*-downregulated ovarian cancer cells

**Fig. 5:**
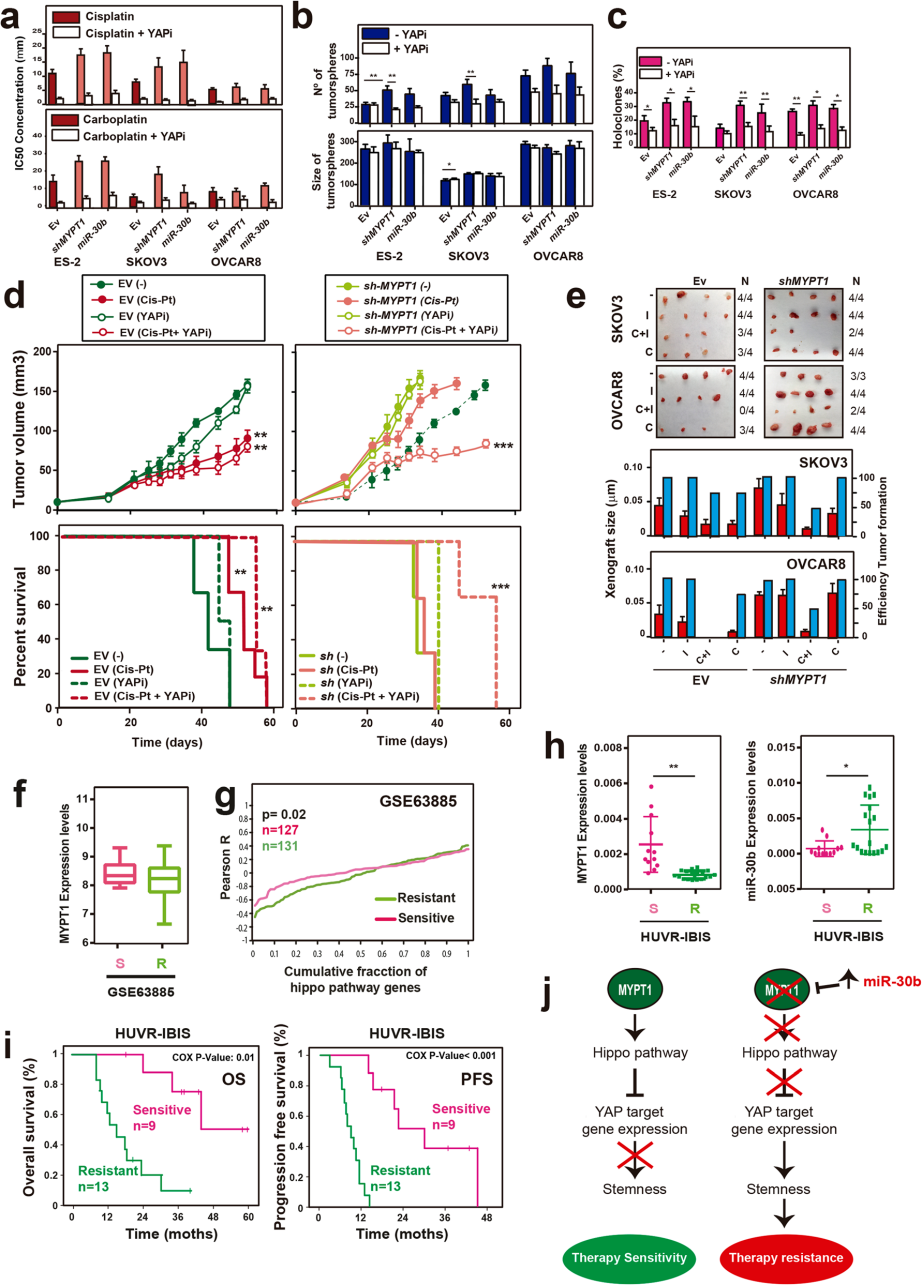
Downregulation of *MYPT*1 increases resistance to platinum treatment by activating the Hippo pathway. **a** Determination of the IC50 for cis-platinum in combination or not with 2 nM of the YAP inhibitor verteporfin (YAPi) in ES-2, SKOV3 and OVCAR8 cells overexpressing *shMYPT1*, miR-30b or EV. **b** Quantification of the number and size of tumorspheres formed in the same cells and conditions than *a*. **c** Percentage of holoclones formed in the same cells and conditions than *a.* **d** Determination of the tumor volume (top) and survival (bottom) after treatment with cisplatin and/or 100 nM YAPi in xenografts of SKOV3 cells expressing *shMYPT1* or EV. **e** Determination of the efficiency of tumor formation and size of xenografts from tumorspheres derived from SKOV3 and OVCAR8 cells expressing *shMYPT1* or EV, treated with saline, cisplatin, 2 nM YAPi or both. **f** *MYPT1* expression in the GSE63885 ovarian cancer patient database. Box plots showing the expression levels of *MYPT1* in ovarian platinum-sensitive (S; pink) or platinum-resistant (R; green) patients. **g** Cumulative distribution of Pearson’s correlation with the Hippo pathway genes from GSE63885. **h** Analysis of the *MYPT1* and miR-30b expression level by RT-qPCR in a cohort of ovarian cancer patients that were sensitive (S; pink) or resistant (R; green) to platinum treatment (HUVR-IBIS). See Additional file 1: Table S4. **i** Kaplan-Meier plots showing overall or progression-free survival in patients who were sensitive (pink) or resistant (green) to platinum treatment in the HUVR-IBIS cohort. **j** Proposed model for how MYPT1 loss induces resistance to treatment with platinum therapy. Briefly, MYPT1 absence leads to deactivation of the Hippo pathway, which in turn favors YAP activation and target gene expression of genes associated to tumor growth and stemness. This increased stemness would be responsible for therapy resistance due to the increase in the CSC population

Finally, we examined whether the activity of the Hippo pathway could be related to the resistance to treatment that is observed in ovarian cancers expressing low levels of *MYPT1* (Fig. 3). To address this possibility, we treated the cells with different doses of cisplatin or carboplatin to determine the IC50 using two different inhibitors that disrupt the interaction between YAP and TEAD transcription factors: peptide 17 and verteporfin. We found that YAP inhibition made ES-2, SKOV3 and OVCAR8 ovarian cancer cells more sensitive to treatment with cisplatin or carboplatin (Fig. 5a and Additional file 3: Figure S7a). Interestingly, YAP inhibition suppressed the higher resistance to both compounds of *MYPT1*-depleted ES-2 and SKOV3 cells, suggesting that resistance can be overcome by repressing Hippo target gene expression.

To assess whether the Hippo-dependent resistance to platinum-derived compounds was linked to the enhancement in stemness upon *MYPT1* depletion, we first analyzed the formation of tumorspheres under verteporfin treatment. We found that YAP inhibition suppressed the increased number of tumorspheres in cells expressing *shMYPT1* or miR-30b (Fig. 5b and Additional file 3: Figure S7b). We also found that YAP inhibition suppressed the increase in holoclones and the decrease in paraclones induced by *MYPT1* downregulation (Fig. 5c and Additional file 3: Figure S7c-d). Altogether, these results suggest that the Hippo pathway mediates the increase in stemness that is caused by the low expression of *MYPT1*, which is responsible for therapy resistance.

To check whether YAP inhibition could suppress therapy resistance mediated by *MYPT1* downregulation in vivo, we generated xenografts with SKOV3 ovarian cancer cells expressing EV or *shMYPT1* and cohorts of 5 mice each were treated with cisplatin, verteporfin or both drugs (Fig. 5d). Consistent with the previous results (Fig. 3e), cisplatin treatment caused a 41% reduction in tumor volume (Fig. 5d), increasing the survival by more than 25% compared to that of the controls (40 vs. 50 days, respectively) in EV-expressing cells. In contrast, cisplatin treatment in *MYPT1*-downregulted cells did not cause a significant effect on either the tumor volume or survival (Fig. 5d). However, combination treatment with cisplatin and verteporfin caused a 51% reduction of tumor volume in xenografts from SKOV3 cells expressing *shMYPT1* (Fig. 5d), reaching similar levels to the xenografts generated from control cells treated with cisplatin. Consistently, survival increased more than 60% with combination treatment with cisplatin and verteporfin (Fig. 5d), and both the efficiency of tumor formation and the final xenograft size were decreased (Fig. 5e). Taken together, these data indicate that the increased YAP activation induced by the depletion of *MYPT1* is responsible for cisplatin therapy resistance in ovarian tumors and that this effect can be reversed by YAP inhibition.

To validate our data in patients, we analyzed the *MYPT1* expression levels in a public ovarian cancer patient database (GSE63885) that contains samples of patients treated with platinum-based chemotherapy (Fig. 5f). We found that resistant patients expressed lower levels of *MYPT1* than sensitive patients, suggesting a role for MYPT1 in therapy resistance. In addition, correlations of Hippo pathway gene expression with *MYPT1* expression were collectively more negative in the resistant patients than in the sensitive ones (Fig. 5g), consistent with an inactivation of the Hippo pathway mediating cisplatin resistance.

Finally, we corroborated these data using a patient sample cohort that was obtained from biopsies of ovarian cancer patients who were sensitive or resistant to treatment with platinum-based chemotherapy. The tumor response to treatment was assessed, identifying nonresponding and responding patients, and the gene expression of the tumors was analyzed (Additional file 1: Table S4). Our results show that *MYPT1* expression in primary samples from platinum-resistant tumors was significantly lower than that in primary samples from platinum-sensitive ovarian tumors (Fig. 5h). Consistent with miR-30b regulating *MYPT1* expression, its expression levels were higher in resistant patients (Fig. 5h). The analysis of overall survival and progression-free survival of this cohort showed that resistant patients had a lower survival probability than sensitive patients (Fig. 5i). Taken together, these results demonstrated that resistance to platinum-derived compounds in ovarian cancer could be induced by the downregulation of *MYPT1* and that this resistance can be suppressed by the inhibition of the Hippo pathway transcriptional co-activator YAP.

## Discussion

Ovarian carcinoma is a highly lethal cancer, mainly due to its late detection and chemoresistance-induced relapse after surgery and/or treatment with platinum-derived compounds [3]. We found that downregulation of the *MYPT1* gene reduced the overall survival of ovarian cancer patients, caused resistance to platinum-based treatment both in vitro and in vivo and led to increased stemness of the tumor cells. This suggests that there is a higher incidence of CSCs with lower *MYPT1* that could account for therapy resistance. Moreover, we showed that this resistance is mediated by the deactivation of the Hippo pathway and that a combination therapy of inhibitors of the Hippo transcriptional co-activator YAP with cisplatin suppressed resistance both in vitro and in vivo.

MYPT1 belongs to the family of myosin phosphatase targeting proteins (MYPT) and functions as a targeting and regulatory subunit of protein phosphatase 1 (PP1). MYPT1 plays a role in the regulation of smooth muscle contraction [11, 12], but other functions of MYPT1 have been recently discovered, such as in migration and cell adhesion [13], cell cycle [14, 15] and development [16]. In addition, a role in cancer has been described for MYPT1, since MYPT1 is inhibited by miR-30d to promote angiogenesis and tumor growth in prostate cancer [41]. Accordingly, we found that *MYPT1* expression is downregulated in human ovarian tumors, and its depletion in ovarian cancer cells and xenograft models promotes tumorigenesis.

We found that *MYPT1* is downregulated in different datasets. We also found that patients with lower *MYPT1* expression showed a significant decrease in the probability of survival in the analyzed databases compared to patients with higher *MYPT1* expression (Fig. 1c). A similarly worse prognosis was identified in patients with higher levels of miR-30b that target MYPT1 (Fig. 1g). These results suggest that *MYPT1* could act as a tumor suppressor in ovarian cancer. Furthermore, in our own patient cohort of resistant and sensitive tumors, we found that patients with tumors resistant to platinum therapy (cisplatin or carboplatin) showed, as expected, worse prognosis correlating with lower levels of *MYPT1* or higher levels of its targeting miR-30b. This finding indicates a clear correlation between *MYPT1* reduction and resistance to tumor therapy in ovary tumors.

Resistance to antitumoral agents, especially cytotoxicity, has been linked to the presence of CSCs in tumors [42, 43]. It is believed that chemotherapy is effective against non-CSC tumor cells but not against CSCs, which are able to initiate new tumor growth after therapy and promote metastasis. Indeed, highly chemoresistant quiescent CSCs have been identified in human ovarian tumors [44]. In this study, we show that *MYPT1* downregulation not only increases the resistance of ovarian cancer cells to platinum-based treatment but also leads to enhanced stem-cell properties. As *MYPT1* is downregulated in many ovarian cancer patients, we propose that the high levels of chemoresistance among these tumors may be due to the increase in the CSC pool due to low levels of *MYPT1*.

MYPT1 has been shown to regulate the Hippo pathway through the dephosphorylation of NF2/Merlin, resulting in YAP/TAZ inhibition [29]. We found that the downregulation of *MYPT1* results in increased NF2/Merlin phosphorylation and, therefore, in a deactivation of the Hippo pathway that leads to increased target gene expression and subsequent tumor growth. Consistently, it has been shown that ILK phosphorylates MYPT1-PP1, leading to its inactivation and promoting tumor progression in breast, colon and prostate cancer cells [30]. In addition, the phosphorylation of MYPT1 by LATS1 in HeLa cells could act as an autoregulatory feedback loop for this pathway [45]. On the other hand, the platelet-induced activation of MYPT1-PP1 has been shown to dephosphorylate YAP/TAZ in ovarian cell lines, thus promoting the expression of the target genes [46]. In our study, we observed that *MYPT1* downregulation resulted in decreased YAP phosphorylation with its subsequent activation, increasing the expression of its target genes. Recently, Zheng and coauthors reported [47] that a small protein of 73 aa codified by a circPPP1R12a promoted the invasion, migration and metastasis in colon cancer also via Hippo signaling [47]. This small protein might act as a dominant negative or peptide interfering with the interaction of MYPT1 (PPP1R12a) with PP1 or NF2. These data taken together illustrate the coding potential of regulators such MYPT1 and the Hippo pathway and the strong regulation of this signaling on stemness and, especially, in cancer resistance. It is worth noting that the Hippo pathway has been related to the tumor microenvironment, so that increased tumor stiffness results in a cancer-associated fibroblast (CAF) phenotype in the non-tumoral stroma. This occurs by the extracellular matrix stiffness inducing YAP activation and this in turn leads to a feed-back loop enhancing the CAF phenotype and reinforcing the matrix stiffness [48–50].

The Hippo pathway has been previously linked to ovarian cancer through YAP, which acts as an oncogene in these tumors [8, 25]. Therefore, YAP targeting may inhibit all tumors with MYPT1 downregulation. To explore this possibility and with the aim of providing a new sensitization therapy, we performed IC50 experiments in vitro, including combination experiments in tumorspheres. Furthermore, we tested this possibility in tumors in vivo. We found that YAP inhibition results in the increased sensitivity of ovarian tumor cells to cisplatin both in vitro and in vivo. These data indicate that the deactivation of the Hippo pathway is responsible for *MYPT1*-induced cisplatin resistance. Interestingly, YAP inhibition also suppresses the increase in stemness features that is induced by *MYPT1* downregulation, thus connecting therapy resistance and ovarian CSCs. Importantly, the in vivo combination treatment with cisplatin and YAP inhibitors is able to decrease tumor growth in xenografts and increase animal survival, suppressing the cisplatin resistance that is induced by *MYPT1* downregulation. These data are supported by the observation that in ovarian cancer patients, resistance is linked to lower *MYPT1* expression and reduced survival.

## Conclusions

We propose a model in which *MYPT1* acts as a tumor suppressor gene in ovarian cancer. MYPT1 activates the Hippo pathway, which normally suppresses YAP-dependent target gene expression and prevents stemness. However, the downregulation of *MYPT1* leads to Hippo pathway inactivation, thereby allowing YAP-dependent target gene expression and increasing cell proliferation, dedifferentiation to a CSC-like state and resistance to platinum-based therapies (Fig. 5j). In these circumstances, YAP inhibition prevents stemness and restores therapy sensitivity. Therefore, *MYPT1* expression could be used as a predictor of the response to treatment in ovarian cancer, allowing the stratification of patients. In addition, these findings have important implications for the treatment of ovarian cancer patients, as they demonstrate the possibility of targeting the Hippo pathway in combination with the use of platinum-derived compounds in patients with low *MYPT1* expression to reduce cancer recurrence and metastasis.

## Supplementary information

**Additional file 1 : Table S1.** Reagents used in this work. **Table S2.** Characteristics of patient public databases used in this study. **Table S3.** CSC markers in OVCAR8 and SKOV3 ovarian tumor cell lines. **Table S4.** Patient Cohort characteristics.

**Additional file 2.** Gene Ontology (GO) analysis of genes whose expression is correlated with that of MYPT1 and gene expression data from Taqman arrays and heatmaps

**Additional file 3 : Figure S1.** Copy number alterations and expression of miR-30b. **Figure S2.** Downregulation of *MYPT*1 decreases Hippo pathway activation. **Figure S3.** Downregulation of *MYPT1* increases tumorigenesis and resistance to platinum in ovarian cancer in vivo and in vitro*.* **Figure S4.** Representative images of MYPT1, NF2 and YAP immunostaining. **Figure S5.** Downregulation of *MYPT1* increases stemness in ovarian cancer. **Figure S6.** CSC surface markers are increased upon *MYPT1* depletion**. Figure S7.** Downregulation of *MYPT*1 increases resistance to platinum treatment by inhibiting the Hippo pathway.

## Data Availability

The datasets used and/or analysed during the current study are available from the corresponding author on reasonable request.

## Abbreviations

CSC: Cancer stem cell
EV: Control cells
FACS: Fluorescence-activated cell sorting
miRNAs: MicroRNAs
