# Supplementary material for: Downregulation of MYPT1 increases tumor resistance in ovarian cancer by targeting the Hippo pathway and increasing the stemness
Source: Mol Cancer. 2020 Jan 11;19:7. doi: 10.1186/s12943-020-1130-z (PMC6954568; doi:10.1186/s12943-020-1130-z)
Supplement: Supplementary file 1 — Additional file 1 : Table S1. Reagents used in this work. Table S2. Characteristics of patient public databases used in this study. Table S3. CSC markers in OVCAR8 and SKOV3 ovarian tumor cell lines. Table S4. Patient Cohort characteristics. [file 12943_2020_1130_MOESM1_ESM.pdf]

dADDITIONAL FILE_1

Muñoz-Galván et al., "Downregulation of *MYPT1* increases tumor resistance in ovarian cancers by targeting the Hippo pathway and increasing the stemness"

Table S1. Reagents used in this work.

**Antibody**	**Dilution**	**Reference**
anti-MYPT1 (D6C1)	1:1000	Cell Signaling #8574S
anti-NF2 [AF1G4]	1:1000	Abcam #ab88957
anti-NF2/Merlin (phosphoS518)	1:1000	Abcam #ab2478
phospho-MST1/2 (T180/T183)	1:200	Abcam #ab76323
phospho-LAST1 (T1079)	1:1000	Cell Signaling #8654S
LAST1	1:1000	Cell Signaling #3477S
YAP	1:1000	Cell Signaling #4912
TAZ	1:1000	Abcam #ab84927
XIAP (H-202)	1:500	SantaCruz#sc-11426
hnRNP C1/C2 (4F4)	1:500	SantaCruz#sc-32308
phospho-YAP (Ser127)	1:1000	Cell Signaling #13008
mAb anti-α-tubulin	1:5000	Sigma 9026
peroxidase-labeled rabbit anti-mouse	1:10000	Amersham
peroxidase-labeled goat anti-rabbit	1:10000	Abcam #6721
CD10-FITC		Myltenyi
CD19-APC		Myltenyi
CD24-PE		Myltenyi
CD34-PE		Myltenyi
CD44-APC		Myltenyi
CD117-APC		Myltenyi
CD133-PE		Myltenyi
CD184-PE		Myltenyi
**Probe**		**Reference**
MYPT1		ThermoFisher#Hs01552899
NANOG		ThermoFisher#Hs04260366
BMI-1		ThermoFisher#Hs00995536
SOX2		ThermoFisher#Hs01053049
OCT4		ThermoFisher#Hs00999632
BIRC5		ThermoFisher#Hs04194392
CTGF		ThermoFisher#Hs01026927
FGF1		ThermoFisher#Hs01092738
GLI2		ThermoFisher#Hs01119974
EPCAM		ThermoFisher#Hs00901885
CD44		ThermoFisher#Hs01075864
MIR30B		ThermoFisher#Hs4427975
GAPDH		ThermoFisher#Hs03929097

Table S2: Characteristics of patient public databases used in this study

	GSE40595 N=77	GSE38666 N=45	GSE14764 N=80
**Age (years)**	0-76	41-78	36-81
**Stage (FIGO 2014)**			
• I		0	7 (8.8%)
• II		7 (15.5%)	1 (1.3%)
• III		18 (40%)	67 (83.8%)
• IV	High grade	0	5 (6.3%)
• n.a.		20 (44.5%)	
**Histology**			
• Serous carcinoma	77(100%)	45 (100%)	67 (83.8%)
• Non serous carcinoma	0	0	10 (12.5%)
• undiff	0	0	3 (3.8%)

Table S3. CSC markers in OVCAR8 and SKOV3 ovarian tumor cell lines

CSC marker	OVCAR8		SKOV3	
(%)	Ev	*shMYPT1*	Ev	*shMYPT1*
CD10	0.2 ± 0.1	0.3 ± 0.1	0.3 ± 0.1	0.6 ± 0.1
CD19	0.3 ± 0.1	0.4 ± 0.1	0.2 ± 0.1	6.5 ± 0.2
CD24	≥ 99%	≥ 99%	≥ 99%	≥ 99%
CD34	0	0	0	0
CD44	≥ 99%	≥ 99%	≥ 99%	≥ 99%
CD117	0.3 ± 0.15	0.4 ± 0.1	0.3 ± 0.1	0.5 ± 0.1
CD133	0.3 ± 0.1	0.4 ± 0.1	0.2 ± 0.1	0.5 ± 0.2
CD184	8.8 ± 0.5	8.6 ± 0.4	0.2 ± 0.1	0.2 ± 0.1

Table S4: Patient Cohort characteristics

	Sensitive N=9 (40%)	Resistant N=13(60%)
**Age (years)**		
• Mean (Rank)	62,0 (34-70)	51,0 (40-67)
**ECOG**		
• 0	7 (77,8%)	5 (38,5%)
• 1	1 (11,1%)	6 (46,2%)
• 2	1 (11,1%)	2 (15,4%)
**Stage (FIGO 2014)**		
• IA	1 (11,1%)	1 (7,7%)
• IC	1 (11,1%)	1 (7,7%)
• IIB	1 (11,1%)	0
• IIIB	1 (11,1%)	1 (7,7%)
• IIIC	4 (44,4%)	8 (61,5%)
• IVA	1 (11,1%)	0
• IVB	0	2 (15,4%)
**Ca 125 (U/ml)**		
• **Diagnosis:** Median (Rank)	194 (31,6-21957)	332 (38-3892)
• **After treatment:** Median (Rank)	11 (6,5-1400)	76,1 (15,5-1862)
**Adjuvant Chemotherapy**		
• No	6 (66,7%)	7 (53,8%)
• Yes	3 (33,3%)	6 (46,2%)
**Treatment**		
• Carbo + Paclitaxel	6 (68%)	12 (92%)
• Carbo + Paclitaxel + beva	3 (32%)	0
• Carbo monoterapia	0	1 (8%)
**Surgery**		
• R0	1 (11,1%)	2 (15,4%)
• R1	2 (22,2%)	2 (15,4%)
• Biobsies	0	2 (15,4%)
• No (incluyen pacientes con cirugía primaria)	6 (66,7%)	7 (53,8%)
**Mejor respuesta a QT adyuvante o 1ª línea**		
• RC	6 (66,7%)	4 (30,8%)
• RP	3 (33,3%)	2 (15,4%)
• EE	0	2 (15,4%)
• PE	0	5 (38,5%)
**Treatment after 1st line**		
• Bevacizumab	3 (33,3%)	0
• Others	0	0
• No	6 (66,7%)	13 (100%)
**Progression disease after treatment**		
• yes	6 (66,7%)	13 (100%)
• No	3 (33,3%)	0
**Platinum free interval (moths)**		
• Mean (Rank)	19 (7-33)	1 (0-5)
**More than 2 lines of treatment**		
• yes	4 (44,4%)	4 (30,8%)
• No	5 (55,6%)	9 (69,12%)
**Status patient in last visit**		
• Live without disease	2 (22,2%)	0
• Live with disease	4 (44,4%)	2 (15,4%)
• Death (all due to disease progression)	3 (33,3%)	11 (84,6%)
**Location primary tumor**		
• Right ovary	2 (22,2%)	2 (15,4%)
• Left ovary	2 (22,2%)	4 (30,8%)
• Bilateral	5 (55,6%)	5 (38,5%)
• Peritoneal	0	2 (15,4%)
**Differentiation**		
• Moderately	1 (11,1%)	1 (7,7%)

• Poor<br>• nd	7 (77,8%)<br>1 (11,1%)	11 (84,6%)<br>1 (7,7%)
**Histology**<br>• Serous carcinoma<br>• Clear cell carcinoma<br>• Others	 7 (77,8%)<br>2 (22,2%)<br>0	 8 (61,5%)<br>3 (23,1%)<br>2 (15,4%)
**Lymphovascular infiltration**<br>• No<br>• Yes<br>• nd	 1 (11,1%)<br>2 (22,2%)<br>6 (66,7%)	 1 (7,7%)<br>1 (7,7%)<br>11 (84,6%)
**BRCA Mutation**<br>• No<br>• Yes<br>• nd	 4 (44,4%)<br>2 (22,2%)<br>3 (33,3%)	 6 (46,2%)<br>0<br>7 (53,8%)
