# Supplementary material for: Downregulation of MYPT1 increases tumor resistance in ovarian cancer by targeting the Hippo pathway and increasing the stemness
Source: Mol Cancer. 2020 Jan 11;19:7. doi: 10.1186/s12943-020-1130-z (PMC6954568; doi:10.1186/s12943-020-1130-z)
Supplement: Supplementary file 3 — Additional file 3 : Figure S1. Copy number alterations and expression of miR-30b. Figure S2. Downregulation of MYPT1 decreases Hippo pathway activation. Figure S3. Downregulation of MYPT1 increases tumorigenesis and resistance to platinum in ovarian cancer in vivo and in vitro. Figure S4. Representative images of MYPT1, NF2 and YAP immunostaining. Figure S5. Downregulation of MYPT1 increases stemness in ovarian cancer. Figure S6. CSC surface markers are increased upon MYPT1 depletion. Figure S7. Downregulation of MYPT1 increases resistance to platinum treatment by inhibiting the Hippo pathway. [file 12943_2020_1130_MOESM3_ESM.pdf]

ADDITIONAL FILE_3

Muñoz-Galván et al., "Downregulation of *MYPT1* increases tumor resistance in ovarian cancers by targeting the Hippo pathway and increasing the stemness"

Figure S1. Copy number alterations and expression of miR-30b. Blox plot showing miR-30b expression levels associated to the copy number alterations observed in patients from the ovarian cancer TCGA database. Statistical differences were analyzed by the Mann-Whitney's *U*-test. ***, P<0.001; ****, P<0.0001.

Figure S2. Downregulation of *MYPT*1 decreases Hippo pathway activation. (**a**) Western blot showing the protein levels of MYPT1, pYAP (S127), YAP and α-tubulin in ES-2, SKOV3 or OVCAR8 ovarian cells expressing *shMYPT1-4* or empty vector (EV). (**b**) Heatmaps showing the expression fold-change of the Hippo pathway genes in ES-2, SKOV3 or OVCAR8 ovarian cells expressing *shMYPT1-4* or miR-30b compared to cells expressing the EV. Hierarchical clustering of the samples is shown. (**c**) Heatmaps showing the z-scores of Hippo pathway gene expression in ES-2, SKOV3 or OVCAR8 cells expressing the EV. Hierarchical clustering of the samples is shown. (**d**) Quantification of MYPT1, NF2 and pNF2 protein levels from western blots in Figure 2h, as well as the phospho-NF2/NF2 ratio. (**e**) Western blot and quantification of the protein levels of MYPT1, YAP and TAZ either in the cytoplasmic or nuclear fractions in ES-2, SKOV3 or OVCAR8 ovarian cells expressing *shMYPT1-4* or EV. Xiap and hnRNP C1/C2 were used as cytoplasmic and nuclear controls, respectively. (**f**) Determination of the methylation status of the *NF2* gene promoter in the SKOV3 or OVCAR8 ovarian cancer cell lines. (**g**) Analysis of the expression by RT-qPCR of several Hippo pathway target genes, including *BIRC5, CTGF, FGF1* and *GLI2*, in SKOV3 or OVCAR8 ovarian cancer cells expressing *shMYPT1-4* or EV.

Figure S3. Downregulation of *MYPT1* increases tumorigenesis and resistance to platinum in ovarian cancer *in vivo* and *in vitro*. (**a**) Quantification of the number of clones in ES-2, SKOV3 or OVCAR8 ovarian cell lines carrying EV (dark green) or *shMYPT-4* (light green). (**b**) Growth curve of ES-2, SKOV3 or OVCAR8 ovarian cell lines carrying EV (dark green) or *shMYPT1-4* (light green), represented as the accumulation of doubling times. (**c**) Determination of the IC50 (concentration of drug necessary to induce 50% of cell death) to platinum-derived drugs in cells overexpressing *shMYPT1-4* or EV. **(d)** Determination of tumor volume and survival after carboplatin treatment of xenografts of SKOV3 cells expressing *shMYPT1-4* or EV. Cohorts of 5 mice each one

were either treated with carboplatin or saline serum once the tumor reached 0.5 cm of diameter, and survival rates were determined. All experiments were repeated at least three times. Data were analyzed using Student's *t*-test. *, P < 0.05; **, P < 0.01; ***, P < 0.001.

Figure S4. Representative images of MYPT1, NF2 and YAP immunostaining in xenografts derived from SKOV3 or OVCAR8 ovarian cells expressing *shMYPT1* or EV. Scale bar: 100 μm.

Figure S5. Downregulation of *MYPT1* increases stemness in ovarian cancer. (**a**) Percentage of paraclones, meroclones and holoclones formed from ES-2, SKOV3 or OVCAR8 ovarian cells expressing *shMYPT1-4* or EV. (**b**) Quantification of the number and size of tumorspheres formed from ES-2, SKOV3 and OVCAR8 cells expressing *shMYPT*1-4 or EV. (**c**) Same as in *b* but from single cells. (**d**) Analysis of the expression of several stemness-associated genes, including *OCT4, NANOG* and *SOX2*, as well as *MYPT1* by RT-qPCR in total extracts and tumorspheres from ES-2, SKOV3 or OVCAR8 ovarian cancer cells expressing shMYPT1-4 or EV. (**e**) Analysis of the expression of several hippo pathway targets genes, including *BIRC5, CTGF, FGF1* and *GLI2*, by RT-qPCR in tumorspheres from ES-2, SKOV3 or OVCAR8 ovarian cancer cells expressing *shMYPT-4* or EV. Average and SD of three independent experiments are shown. Data were analyzed using Student's *t*-test. *, P < 0.05; **, P < 0.01; ***, P < 0.001.

Figure S6. CSC surface markers are increased upon *MYPT1* depletion. Representative plots of CD10+, CD133+ and CD19+ CSC surface markers analyzed by FACS in ES-2, SKOV3 or OVCAR8 ovarian cancer cells expressing *shMYPT1*, miR-30b or EV.

Figure S7. Downregulation of *MYPT*1 increases resistance to platinum treatment by inhibiting the Hippo pathway. (**a**) Determination of the IC50 to platinum drugs in combination with 2 nM of the YAP inhibitor verteporfin (YAPi) in ES-2, SKOV3 or OVCAR8 expressing *shMYPT1-4* or EV. (**b**) Quantification of the number and size of tumorspheres formed from ES-2, SKOV3 and OVCAR8 ovarian cells expressing *shMYPT1-4* or EV, treated or not with YAPi. (**c**) Percentage of paraclones, meroclones and holoclones formed from ES-2, SKOV3 or OVCAR8 ovarian cells expressing *shMYPT1, miR-30b* or EV, treated or not with YAPi. (**d**) Percentage of paraclones,

meroclones and holoclones formed from ES-2, SKOV3 or OVCAR8 ovarian cells expressing *shMYPT1-4* or EV, treated or not with YAPi.

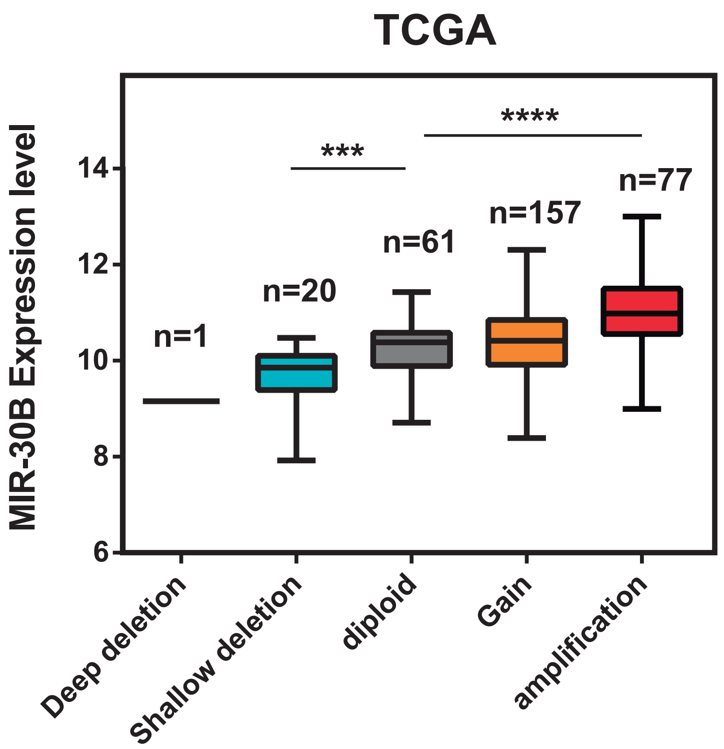
TCGA
MIR-30B Expression level
14
12
10
8
6
***
****
n=1
n=20
n=61
n=157
n=77
Deep deletion
Shallow deletion
diploid
Gain
amplification

Figure S1

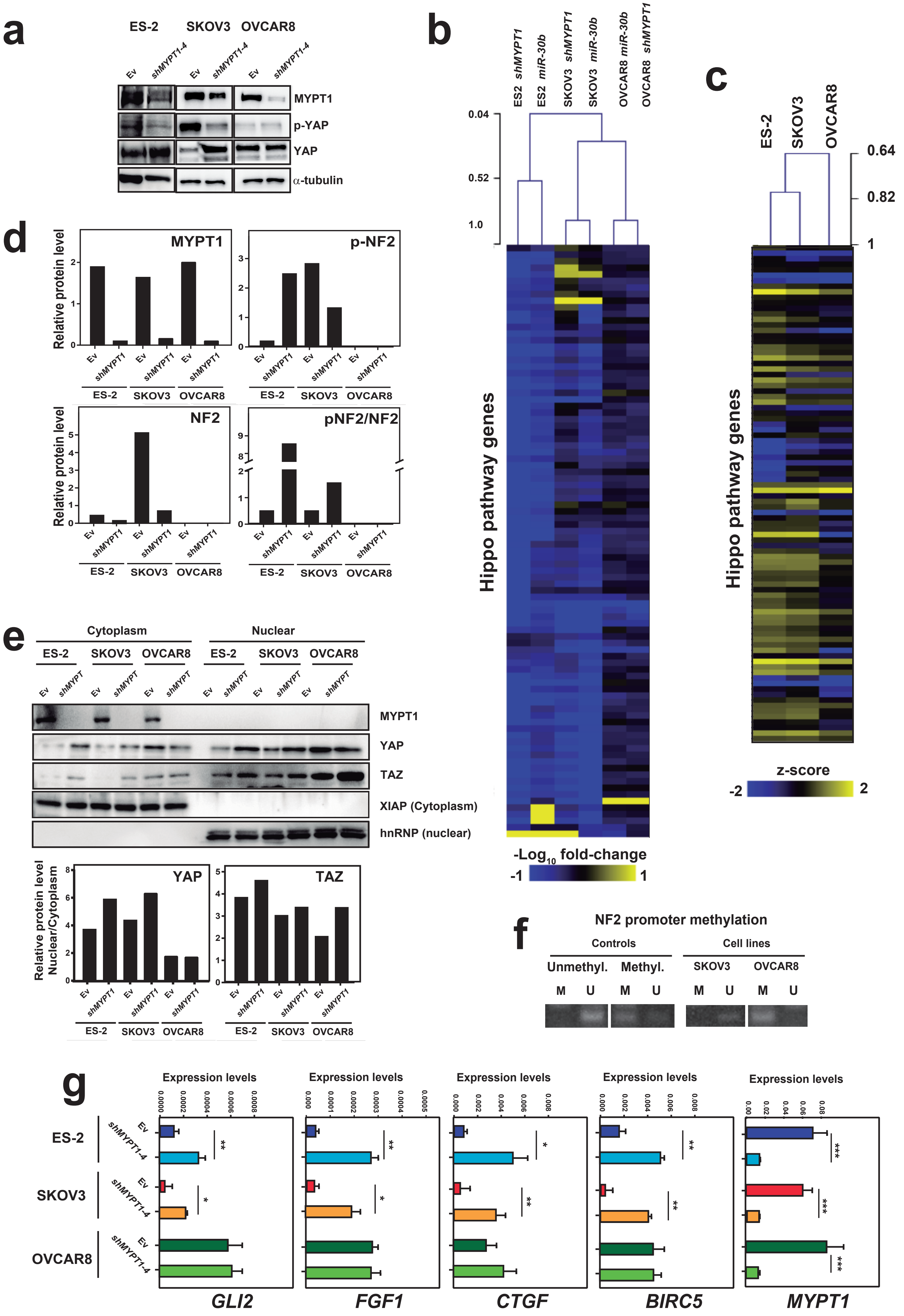

a
ES-2
SKOV3
OVCAR8
Ev
shMYPT1-4
MYPT1
p-YAP
YAP
α-tubulin
b
ES2 shMYPT1
ES2 miR-30b
SKOV3 shMYPT1
SKOV3 miR-30b
OVCAR8 miR-30b
OVCAR8 shMYPT1
Hippo pathway genes
-Log10 fold-change
c
z-score
d
Relative protein level
MYPT1
p-NF2
NF2
pNF2/NF2
e
Cytoplasm
Nuclear
shMYPT
TAZ
XIAP (Cytoplasm)
hnRNP (nuclear)
Relative protein level Nuclear/Cytoplasm
f
NF2 promoter methylation
Controls
Cell lines
Unmethyl.
Methyl.
M
U
g
Expression levels
GLI2
FGF1
CTGF
BIRC5

Figure S2

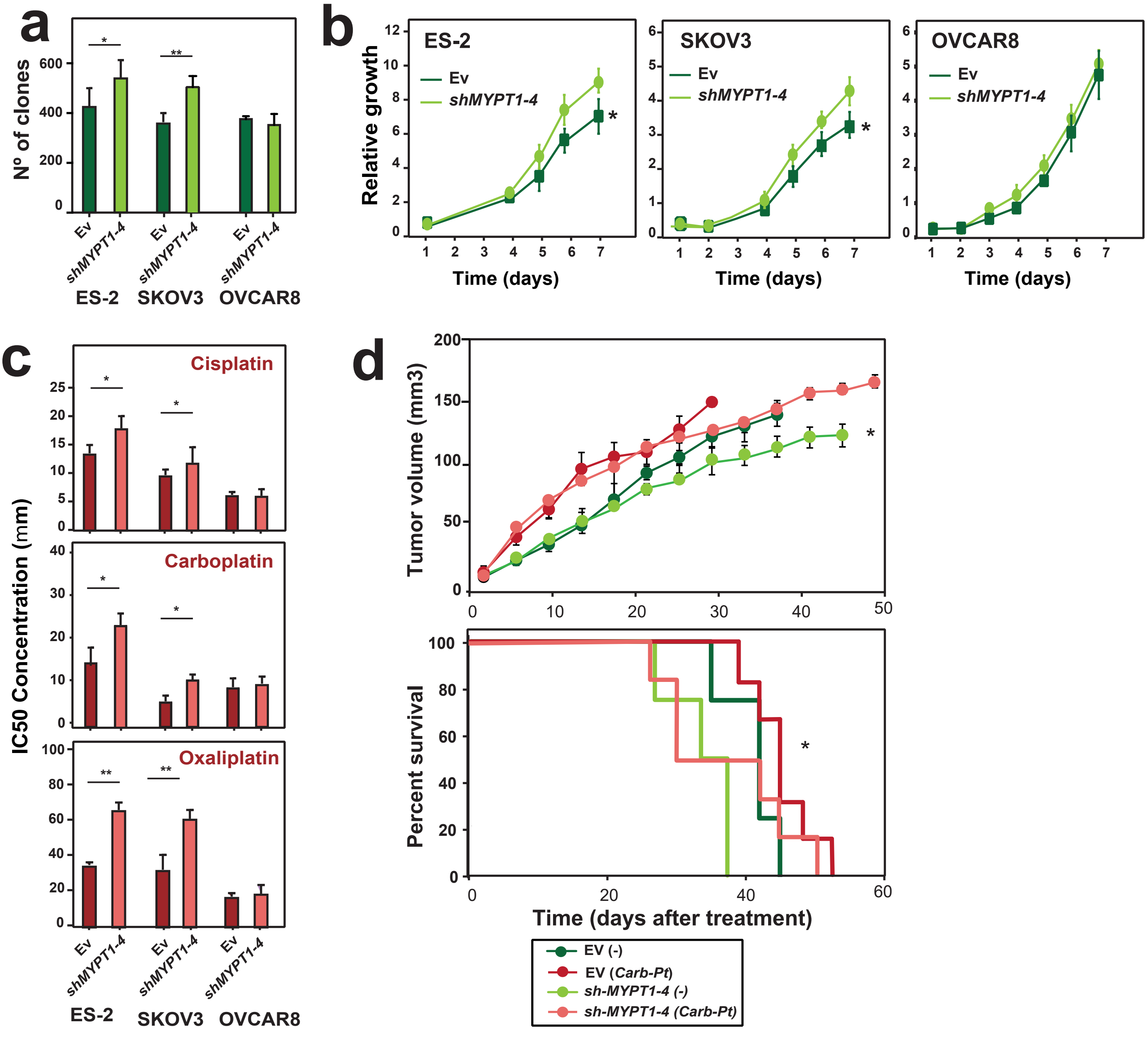

a
Nº of clones
ES-2
SKOV3
OVCAR8
Ev
shMYPT1-4
b
Relative growth
ES-2
SKOV3
OVCAR8
Ev
shMYPT1-4
Time (days)
c
IC50 Concentration (mm)
Cisplatin
Carboplatin
Oxaliplatin
d
Tumor volume (mm3)
Percent survival
Time (days after treatment)
EV (-)
EV (Carb-Pt)
sh-MYPT1-4 (-)
sh-MYPT1-4 (Carb-Pt)

Figure S3

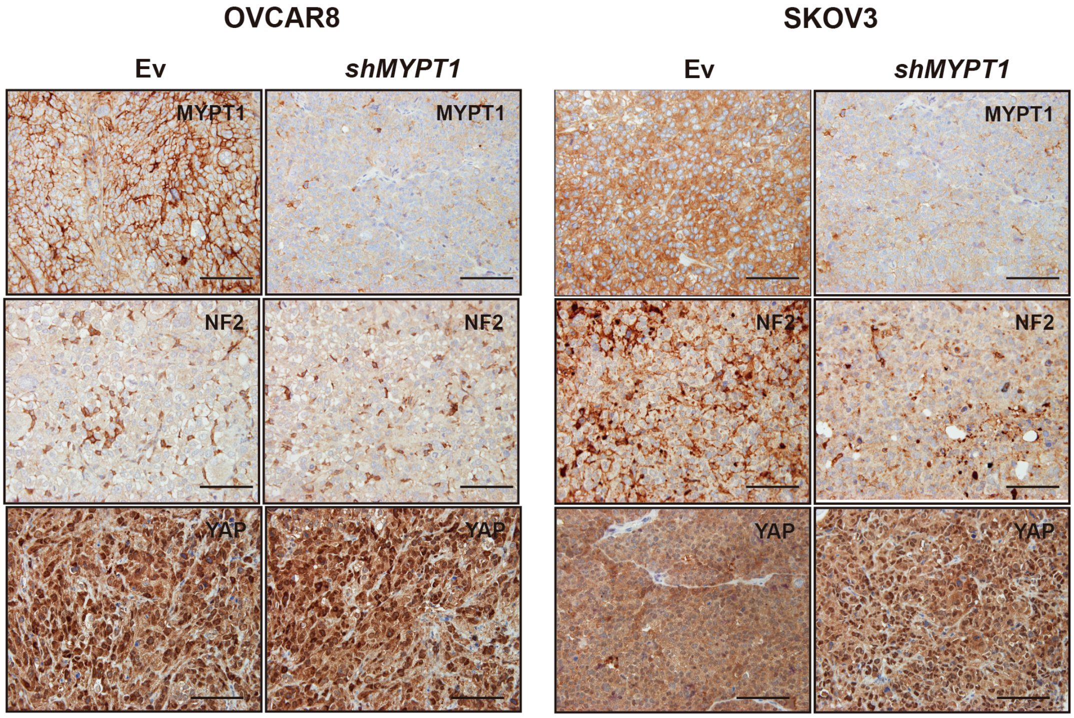
OVCAR8
SKOV3
Ev
shMYPT1
Ev
shMYPT1
MYPT1
MYPT1
MYPT1
MYPT1
NF2
NF2
NF2
NF2
YAP
YAP
YAP
YAP

Figure S4

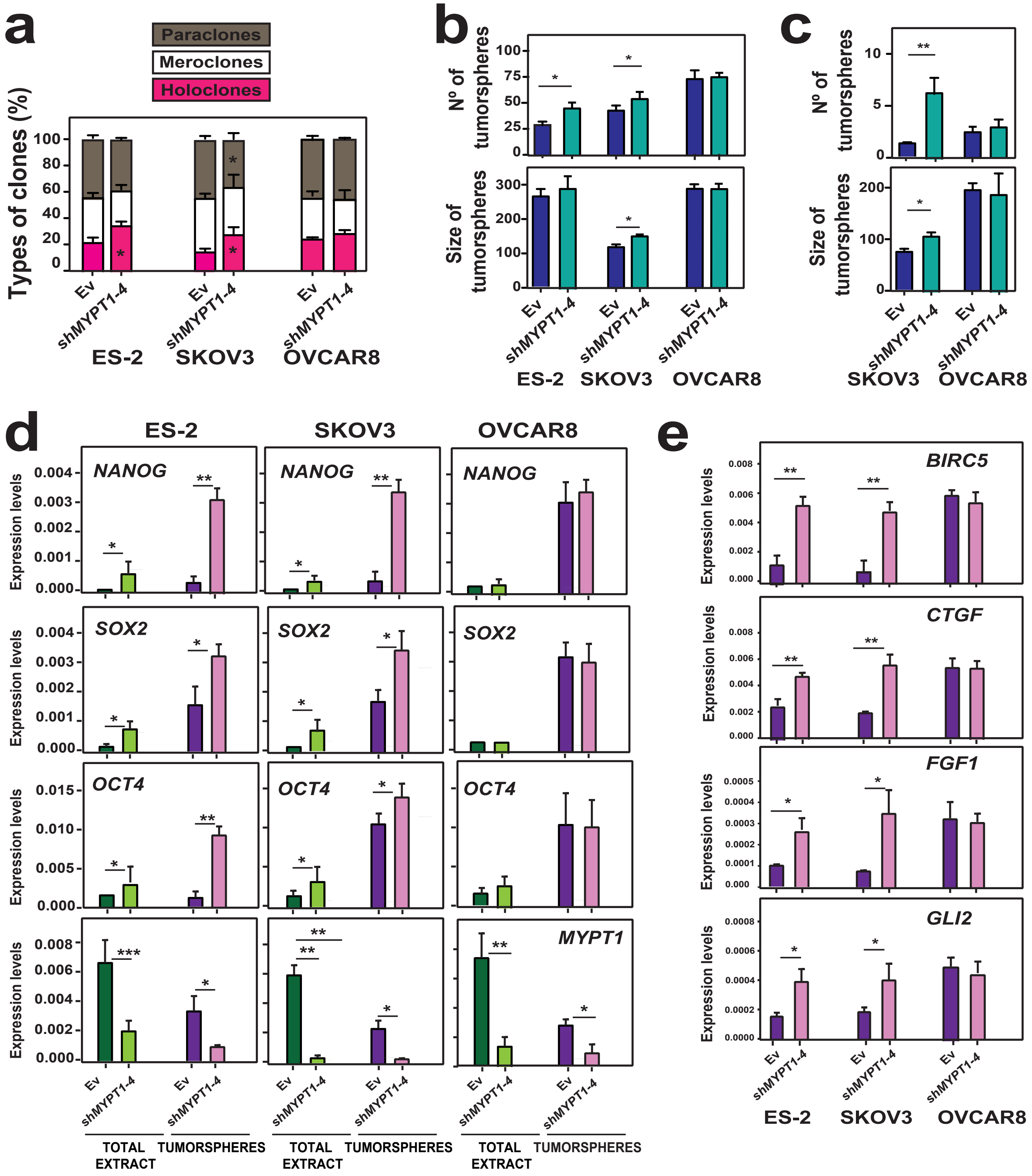
a
Paraclones
Meroclones
Holoclones
Types of clones (%)
Ev
shMYPT1-4
ES-2
SKOV3
OVCAR8
b
Nº of tumorspheres
Size of tumorspheres
c
d
NANOG
SOX2
OCT4
MYPT1
Expression levels
TOTAL EXTRACT
TUMORSPHERES
e
BIRC5
CTGF
FGF1
GLI2

Figure S5

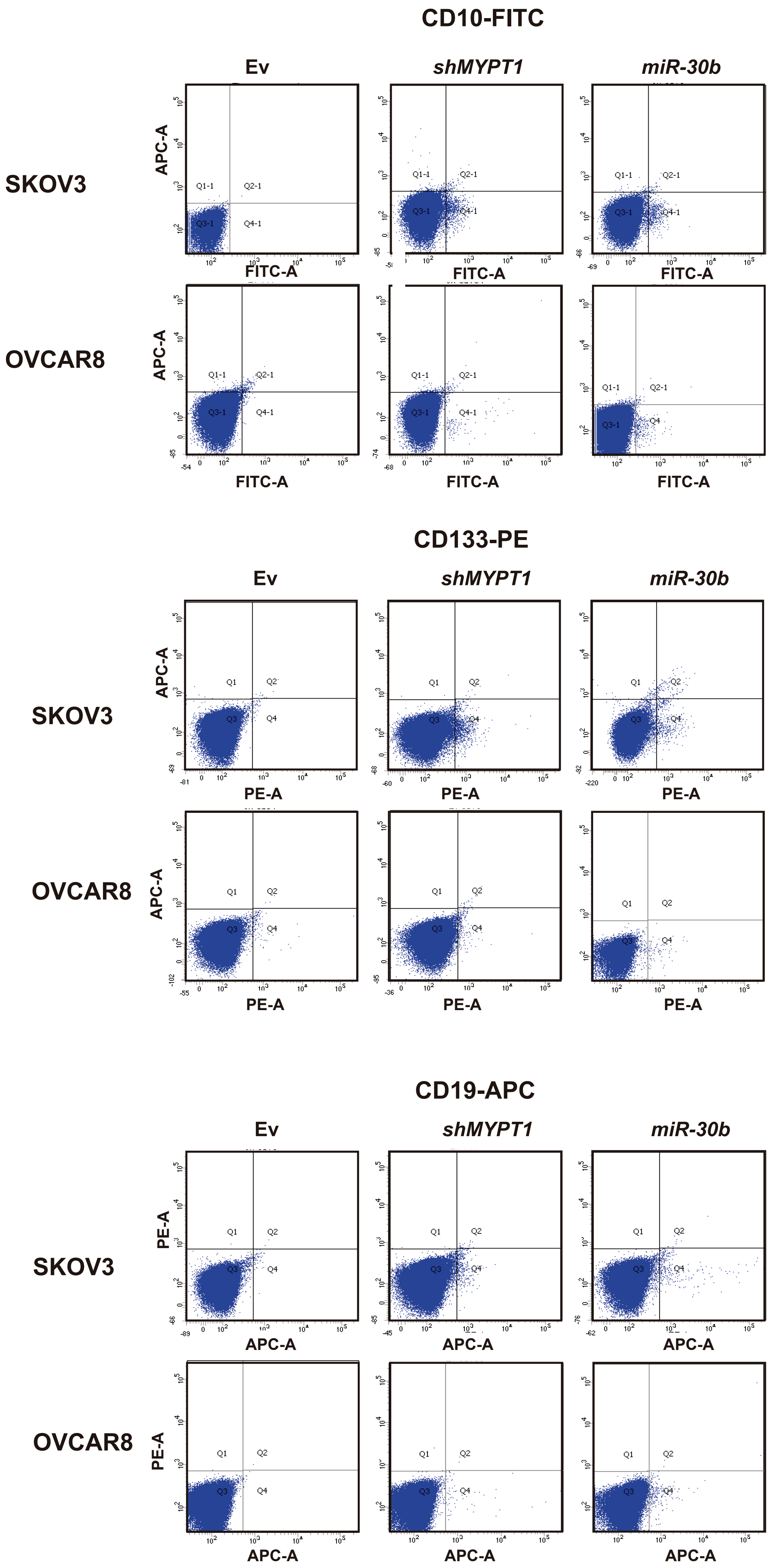
CD10-FITC
Ev
shMYPT1
miR-30b
SKOV3
OVCAR8
APC-A
FITC-A
Q1-1
Q2-1
Q3-1
Q4-1
CD133-PE
Ev
shMYPT1
miR-30b
SKOV3
OVCAR8
APC-A
PE-A
Q1
Q2
Q3
Q4
CD19-APC
Ev
shMYPT1
miR-30b
SKOV3
OVCAR8
PE-A
APC-A
Q1
Q2
Q3
Q4

Figure S6

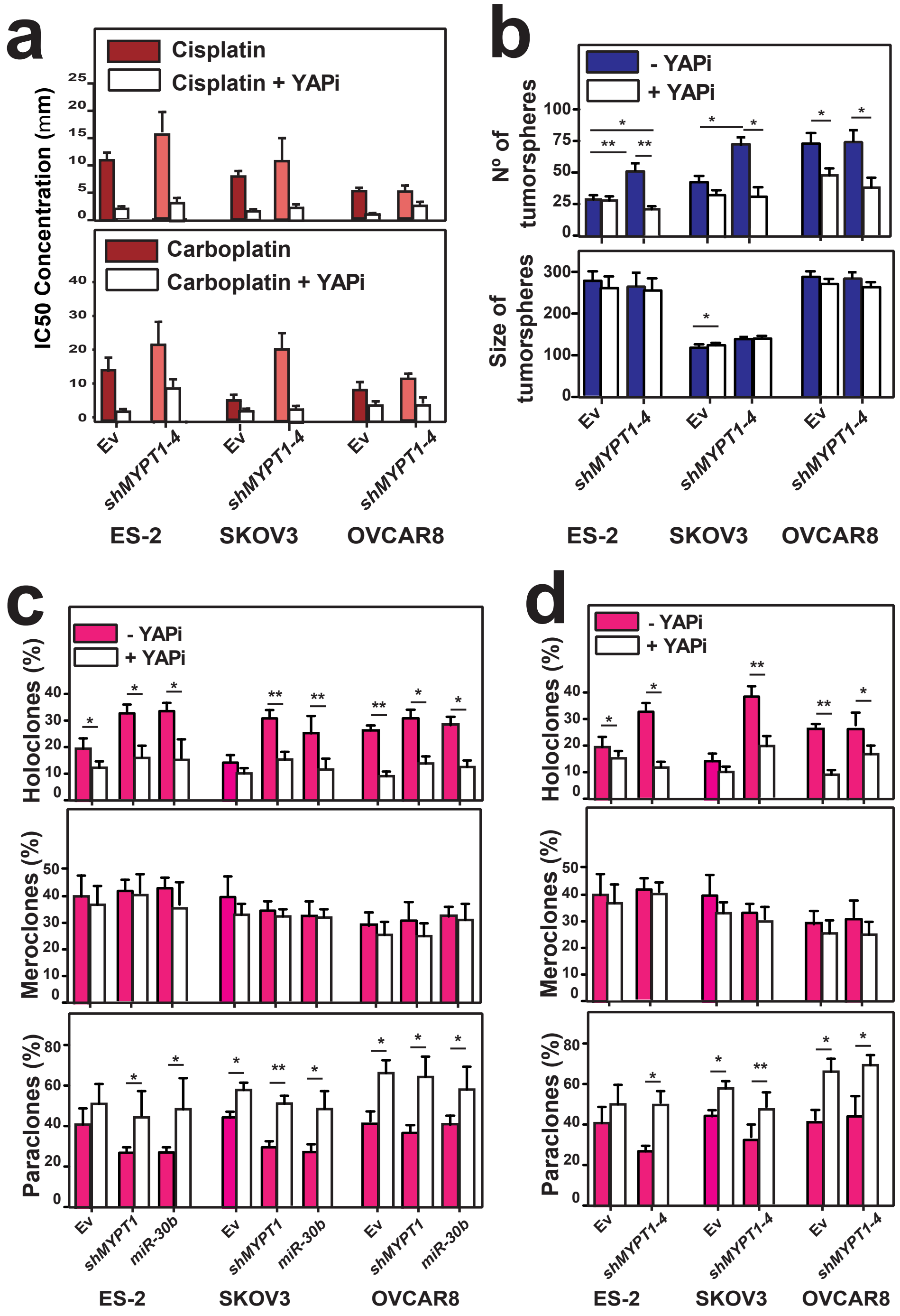
a
Cisplatin
Cisplatin + YAPi
Carboplatin
Carboplatin + YAPi
IC50 Concentration (mm)
Ev
shMYPT1-4
ES-2
SKOV3
OVCAR8
b
- YAPi
+ YAPi
Nº of tumorspheres
Size of tumorspheres
c
Holoclones (%)
Meroclones (%)
Paraclones (%)
shMYPT1
miR-30b
d

Figure S7
